# The German translation of the Oxford utilitarianism scale: Validation and the impact of the Covid-19 pandemic on the observations

**DOI:** 10.1371/journal.pone.0335215

**Published:** 2025-10-27

**Authors:** Aistė Ambrasė, Malte Hendrickx, Melina Grahlow, Hong Yu Wong, Birgit Derntl

**Affiliations:** 1 Department of Psychiatry and Psychotherapy, Women’s Mental Health and Brain Function, Tübingen Center for Mental Health (TüCMH), University of Tübingen, Tübingen, Germany; 2 Philosophy Department, University of Michigan, Ann Arbor, United States of America; 3 Werner Reichardt Centre for Integrative Neuroscience, University of Tübingen, Tübingen, Germany; 4 Department of Philosophy, University of Tübingen, Tübingen, Germany; 5 German Center for Mental Health (DZPG), Partner Site Tübingen, Tübingen, Germany; 6 LEAD Research School and Graduate Network, University of Tübingen, Tübingen, Germany; Islamic Azad University Ahvaz Branch, IRAN, ISLAMIC REPUBLIC OF

## Abstract

The study of utilitarian inclinations is probably the most experimentally investigated aspect of morality. The Oxford Utilitarianism Scale has been developed to provide a self-report tool for reliable measurement of utilitarian views while addressing serious methodological issues with previous measures. In this study, we have translated and validated a German version of the Oxford Utilitarianism Scale (OUS-DE). The scale consists of two subscales: Impartial Beneficence (IB-DE) and Instrumental Harm (IH-DE). We conducted a procedure in a general German sample (N_S1_ = 378, 243 women, M_age_ = 25.37) before the Covid-19 pandemic. A confirmatory factor analysis demonstrated a good fit of a two-factor model for OUS-DE, while internal consistency and construct reliability were acceptable. Both in the pre-pandemic and the post-pandemic sample (N_S2_ = 348, 206 women, M_age_ = 24.61) we found a sex/gender difference, with women scoring significantly higher in the IB-DE subscale than men. We also found that the mean agreement with the IB-DE subscale decreased after the pandemic. In a separate third sample (N_S3_ = 39, 19 women, M_age_ = 23.72), we observed an inverse U-shape relationship between moral behavior related to quarantine requirements and the IH-DE subscale, as measured during the peak pandemic restrictions in late 2020. Repeated OUS-DE measurement in this sample showed stability in responders’ utilitarian beliefs post-pandemic. In sum, OUS-DE is the first available measurement of utilitarian inclinations in German. The scale will enable further research on how utilitarian preconceptions affect behavior in German-speaking populations.

## Introduction

Utilitarianism is a moral theory that holds that the morally correct action is the one that brings about the most good for the greatest amount of people [1–3]. As a consequentialist theory, it is often distinguished from other moral theories by its focus on the consequences of actions (as opposed to, e.g., intentions, rights, or virtues). Due to Utilitarianism’s commitment to intriguing departures from commonsense morality, agreement with utilitarian principles in the general population has been extensively studied in psychology and neuroscience [4–6].

Utilitarianism deviates from the commonsense morality in two important ways. First, in contrast to the requirement of non-interference in commonsense morality, e.g., “not to harm”, “not to steal” [7–11], Utilitarianism controversially instructs one to use harm in an instrumental way in situations where the aggregate outcome would outweigh the cost. For example, it may be morally required to sacrifice a single bystander to divert a trolley racing toward a group of five [12].

Utilitarianism, like commonsense morality, posits a moral obligation to act beneficially towards those in need [13]. However, in a second radical departure from commonsense morality, Utilitarianism advocates for a radically impartial stance in beneficence. It rejects preferential consideration for individuals based on their relation to or distance from the moral agent [14]. For example, Utilitarianism would require choosing a charity based on objective needs rather than national preferences. The departures from commonsense morality with regard to instrumental harm (IH) and radically impartial beneficence (IB) suggest a 2-D model of utilitarian psychology [15].

While Utilitarianism has received more attention in psychology and neuroscience than any other moral theory, its measurement has typically relied on a caricaturized view of Utilitarianism. Agreement with Utilitarianism was operationalized only in terms of agreement of subjects to sacrifice the wellbeing and life of others in moral dilemmas. This led to a situation where both true moral utilitarians and antisocial individuals are classified as utilitarian [16–20]. Utilitarianism is not well captured by an exclusive focus on IH [21]. In fact, IB is considered a distinctive feature of all consequentialist theories, of which Utilitarianism is the most prominent [22].

As a self-report measure, the Oxford Utilitarianism Scale (OUS; [23]), aims to address this problem by representing both IB and IH, thus assessing personal agreement with the main principles of utilitarian moral theory. The questionnaire comprises two subscales: Impartial Beneficence (IB, 5 items) and Instrumental Harm (IH, 4 items) representing the two tenets that distinguish Utilitarianism from other moral views. The two subscales are independent and dissociable as they show no consistent association with each other. Agreement to both is necessary to infer that the moral agent is indeed guided by Utilitarianism and not another moral theory. The two subscales can also be used separately when investigating associations between the two constructs and individual differences as well as other moral inclinations [23].

In the original OUS validation study [23] and further studies on utilitarian morality [24–26] positive weak to moderate correlations between agreement with the original OUS or either of its subscales and agreement with utilitarian choice in moral dilemmas emerged, indicating content validity of the scale. This evidence was reported in sacrificial dilemmas [24,25], indicating that the measurement generally tracks utilitarian attitudes towards IH, and in impartiality dilemmas [26], showing that the whole IB subscale can capture the impartiality principle well.

Since its publication, OUS has proven to be a valuable research tool in assessing utilitarian inclinations in the general population as well as specific specialist groups such as healthcare professionals [27,28]. OUS has been translated and validated into Turkish [29], French [30], and Spanish [31]. Its structural validity was assessed in Chinese, French, German, Greek, Hungarian, Italian, Polish, Portuguese, Romanian, Russian, Serbian, Slovak, and Turkish languages [32]. Most translated and validated versions of OUS confirm the two-factor solution. However, in some languages the translations resulted in a different factor structure [32], indicating either that the translations should be revised and further validation procedures carried out to confirm the two-factor construct of Utilitarianism or that cultural influences should be considered.

### Associations between utilitarian inclinations and other moral dispositional traits.

It’s focus on beneficence and minimization of the suffering when harm is involved places Utilitarianism in the moral domain of Harm/Care. Moral domains are sets of morally relevant issues, such as care and harm, fairness, loyalty, authority, sanctity and purity, and they can be measured by the Moral Foundations Questionnaire [33,34]. In a US study, it has been shown that extraordinary altruists highly endorse IB and are more concerned with the Harm/Care than regular controls [35]. In a Polish study, agreement to Harm/Care mediated the significant relationship between religiosity and IB, showing that in different countries cultural and individual differences might be uniquely affecting people’s utilitarian inclinations.

Concern for care also links Utilitarianism to empathy. In moral philosophy, it has been assumed that empathy, the understanding and reception of otherness, is necessary for care behaviors [36,37]. It has been shown that individuals who exhibit higher empathic concern endorse IB to a greater extent and IH to a lesser extent [15,23,30,38]. This pattern of associations between the two components of Utilitarianism has been also demonstrated in other-benefiting altruistic tasks and moral dilemmas where Utilitarianism requires IH [39–41]. However, the relationship between OUS and its subscale scores with empathy were reversed in the Turkish validation of the OUS [29], indicating again that more research is required. As religiosity fosters beneficent behaviors, a positive association between frequency of religious practice and the IB subscale has been found [23,42], while no associations were established between religiosity and behavior during sacrificial harm moral dilemmas [43].

Some authors have claimed that utilitarians are 1) more morally competent because utilitarian choices are based on deliberation processes rather than intuition or emotion [44–46] and that 2) their choices sometimes are driven by higher action-taking tendencies or lower aversion to harm, rather than a true endorsement of utilitarian principles [39,47–51]. OUS has not yet been compared to such constructs. Doing so might provide novel insight into utilitarian psychology.

### Associations between utilitarian inclinations and sociodemographic variables.

The current state of research on OUS indicates that sociodemographic differences are associated with consistent individual differences in agreement with utilitarian views. Some authors claim that socialization, culture, and biological sex differences lead to different moral inclinations [50,52,53]. For example, some report that women more strongly internalize their moral beliefs, affecting self-evaluations and moral behavior [54,55]. In the original OUS validation study [23], authors found a trend that men endorsed IH to a greater extent than women, which accords with previous findings in sacrificial moral dilemmas [50,51,56–59] and translation studies of OUS [31]. A higher endorsement of IB has been also found in women [31]. This is similar to studies assessing a related construct, altruism, with women more inclined on average to choose altruistic options than men [60–62].

Moral inclinations also differ between different age groups, even in adults [63–67]. Agreement with IB was found to increase with age in China, Spain, and Chile [31,68]. This pattern is consistent with age effects in altruism [69]. However, in a Romanian sample, younger adolescents scored higher on IB than their older underaged counterparts, indicating that the age effect might not be linear [70]. Regarding IH, agreement to this subscale increased with age in China [68] but decreased in Spain and Chile [31]. A consistent age effect has been demonstrated in utilitarian choices during moral dilemmas, where older adults endorse IH to a lower degree than younger adults [66,71]. The heterogeneity of results on age effects in assessing utilitarian inclinations with OUS indicates that country-related age effects should be investigated.

### Effects of Covid-19 pandemic on utilitarian inclinations.

During the Covid-19 pandemic, which started in March 2020 and ended in May 2023 [72], many countries adopted public pandemic policies based on utilitarian principles [73–75]. Germany’s strict pandemic regulations provide ample examples: group/society’s interests were prioritized over interests of individuals, cost/benefit analyses were based on the principle of the greater good in political decisions, and impartiality principles were applied in medical care [76,77]. In daily life, individuals were exposed to personal moral dilemmas, in which the utilitarian option was both personally and socially beneficial [78,79].

This increased exposure to situations where utilitarian choices brought about the largest benefit to society might have altered people’s perceptions of Utilitarianism. Previous research has shown that individuals revise their moral beliefs when their judgments run counter to majority opinion [80] or when imagining a situation in which a proposed contradictory action would be moral [81]. Furthermore, when moral situations are presented in a practical context, as was often the case during the pandemic, individuals tend to be more accepting of moral solutions, even when these solutions contradict their moral views [82]. Taking them together, investigating the effects of Covid-19 pandemic on agreement with OUS and its subscales would provide a valuable addition to research on stability of moral beliefs.

### Associations between utilitarian inclinations and utilitarian behavior.

As OUS measures individuals’ self-reported moral beliefs, a question arises whether the measurement can predict real-life utilitarian behavior. Previous research reported that general moral values and beliefs are only weakly associated with actual moral behavior [83]. It has been shown that the association between moral inclinations and the corresponding moral behavior is mediated by co-occurrent moral emotions, moral courage, and surrounding context [84–87]. However, the OUS items are highly specific and criterion-matched to target the distinctive features of Utilitarianism, and methodological research shows that such measures have higher predictive validity than measurements targeting broader constructs [88]. In line with the specificity criterion, the Covid-19 pandemic presented a prime opportunity to study the degree to which OUS predicts moral behavior. During the pandemic, individuals were expected to follow quarantine rules, designed to minimize suffering and benefit a greater number of people while demanding personal sacrifices [78]. Therefore, it is possible that the OUS would have had stronger associations with behavior during the pandemic, as the pandemic provided a conceptually corresponding context.

### Current studies

Here we report three studies which measured utilitarian inclinations using OUS. The primary aim of Study 1 was to provide a validated German translation of OUS questionnaire (OUS-DE). Our second aim was to investigate the potential influence of demographic variables, such as age, sex/gender, and religiosity, as well as effects of personality and character differences on agreement with OUS-DE, reported in Study 2. Additionally, as the circumstances allowed, in Study 2 we also intended to investigate the effects of the Covid-19 pandemic on the agreement with OUS-DE, comparing observations from two samples collected before and after the pandemic. Finally, in Study 3 we sought to examine the stability of utilitarian inclinations in the same sample with repeated measures during and after the pandemic as well as studying the relationship between utilitarian moral beliefs and self-reported moral behavior and judgement during the pandemic.

## Study 1: Translation and validation of the German Oxford Utilitarianism Scale

The aim of this study was to validate the German translation of the OUS (OUS-DE). We have implemented a validation procedure similar to that done for the original OUS study [23]: we examined the factor structure with confirmatory factor analyses (CFA), assessed the internal consistency, and the split-half reliability of OUS-DE. To establish criterion-related validity of the scale, we implemented the same procedure as the original study by using moral dilemma scenarios related to sacrificial harm and greater good, and self-reported agreement with Utilitarianism.

We hypothesized that a two-factor OUS-DE would reach comparable goodness-of-fit as the original English OUS, and that each of the subscales will show good to excellent model fit in CFA. We expected positive but weak correlations between the two subscales and a strong correlation between the score of each of the subscales and the overall OUS-DE score. OUS has already been translated and validated for use in the Turkish [29], French [30] and Spanish [31] languages, and the CFAs in those studies indicated an excellent two-factor model fit. However, structural validity of a different German translation of OUS was not comparable to the original scale in English language and the scale did not meet configural invariance, indicating that the factorial structure of that German translation was not stable across multiple groups [32]. This prompts the need for another translation and validation study.

### Materials and methods

#### Sample description.

Five hundred five participants completed an online validation survey from 2019-05-14 to 2019-07-14. One hundred twenty-seven participants were excluded from further analyses: 21 participants reported a mother-tongue other than German, two participants failed two attention checks included in the survey, 20 participants completed the survey faster than the predetermined minimal time limit (< 20 minutes), one participant indicated non-binary gender, 70 participants self-reported a mental disorder diagnosis, and 13 participants were identified as outliers in univariate and multivariate outlier analyses (for more details, please see Results). Therefore, the final sample consisted of 378 participants (243 women, M_age _= 25.37, SD = 6.52, for sociodemographic characteristics, please see Table 1).

**Table 1:** Sociodemographic Characteristics of the final sample.

Baseline characteristic	Sample (N = 378)	
	n	%
*Sex/gender*		
Women	243	64,3%
Men	135	35,7%
*Age (years)*		
18-20	79	20,9%
21-25	171	45,2%
26-30	71	18,8%
31-35	20	5,3%
36-40	14	3,7%
41-45	23	6,1%
*Highest education level*		
No completed secondary education	2	0,5%
Secondary	4	1,1%
General (high school)	121	32,0%
At least one higher education degree	251	66,4%
*Religion*		
Christianity	204	54,0%
Judaism	2	0,5%
Islam	7	1,9%
Buddhism	1	0,2%
Agnosticism	48	12,7%
Atheism	103	27,3%
Other	13	3,4%
*Political ideology*		
Marxist/socialist	29	7,7%
Liberal	82	21,7%
Ecologist	108	28,6%
Social democratic	127	33,6%
Conservative	29	7,7%
Nationalistic	3	0,8%

#### Materials

##### Oxford utilitarianism scale.

The Oxford Utilitarianism Scale (OUS) consists of two subscales, i.e., Impartial Beneficence (IB, 5 items) and Instrumental Harm (IH, 4 items), comprised of 9 items in total [23]. The scale is scored on a Likert scale from 1 (strongly disagree) to 7 (strongly agree). Greater scores reflect a greater utilitarian inclination, that is, greater implicit agreement with main principles of utilitarian moral theory. The IB subscale measures the positive dimension of Utilitarianism concerning with duties of beneficence (3 items), treating immoral acts and failures to act morally (omissions) as equally morally wrong (1 item), and, importantly, moral impartiality (1 item). Moral impartiality is an important principle in utilitarian moral theory requires an individual to treat other moral agents equally, despite their social closeness [23]. The IH subscale assesses moral appropriateness of instrumental harm, i.e., harm to somebody used as a collateral to save or benefit a greater number of people (3 items) and short-term political oppression to ensure well-being of the citizens (1 item). Together, the two subscales measure general agreement with a utilitarian moral view and an overall agreement score can be calculated. On the other hand, the two subscales can be measured and their relationships with other measurements assessed separately, i.e., they are dissociable as measurements.

##### Moral dilemma scenarios.

Six moral dilemma scenarios – three sacrificial harm moral dilemmas that capture participants’ behavior in applying instrumental harm for the greater good, and three greater good moral dilemmas that capture participants’ self-sacrificial and impartial behavior – originally used in the validation study by Kahane [23] were used to measure utilitarian inclinations during moral decision-making. Study participants had to evaluate the moral appropriateness of the proposed solution to the sacrificial harm dilemmas by using a scale from 1 (not at all wrong) to 7 (absolutely wrong), with 1 indicating fully utilitarian judgment and 7 indicating fully non-utilitarian judgment. In greater good dilemmas the scoring was reversed, using a scale from 1 (absolutely wrong) to 7 (not at all wrong), with 1 indicating fully non-utilitarian judgment and 7 indicating fully utilitarian judgment.

##### Self-reported utilitarianism.

Participants self-reported whether they considered their view to be utilitarian after reading a paragraph, explaining the theoretical moral commitments of utilitarian and deontological moral theories on a scale from 1 to 10 (low score for deontological view, high score for utilitarian view). The paragraph on utilitarian moral theory was provided by the authors of the original OUS validation [23].

##### Procedure.

The Ethics commission of the Medical Faculty at the University of Tübingen approved the online survey (“Validation of German translation of Oxford Utilitarianism Scale”, project number 839/2018BO2).

The OUS and the validation dilemma scenarios as well as Utilitarianism description for self-reported agreement were translated to German by one of the authors, who specializes in moral philosophy and is a native speaker of German. Two independent English native speakers (one from the field of philosophy and one with non-philosophical background) with a very good knowledge of the German language performed back-translation. Any inconsistencies in translation were assessed by the authors of the study. Overall, the German translation of the OUS (OUS-DE) provided a good understanding of the questionnaire items as only minor inconsistencies in wording in the back-translations were observed. Original and translated versions of the questionnaire are depicted in Table 2.

**Table 2:** English and German versions of OUS.

Subscale	Item No.	Item in English and German
Impartial Beneficence	IB-1	EN: From a moral perspective, people should care about the well-being of all human beings on the planet equally; they should not favor the well-being of people who are especially close to them either physically or emotionally. DE: Unter moralischen Gesichtspunkten sollten sich Menschen gleichermaßen um das Wohlergehen aller Menschen auf dem Planeten sorgen. Sie sollten das Wohlergehen der Personen, die ihnen physisch oder emotional nahe stehen, nicht bevorzugen.
	IB-2	EN: From a moral point of view, we should feel obliged to give one of our kidneys to a person with kidney failure since we don’t need two kidneys to survive, but really only one to be healthy. DE: Von einem moralischen Standpunkt aus gesehen sollten wir uns verpflichtet fühlen, eine unserer Nieren an eine Person mit Nierenversagen zu geben. Denn wir brauchen nicht zwei Nieren um zu überleben, sondern nur eine, um gesund zu sein.
	IB-3	EN: If the only way to save another person’s life during an emergency is to sacrifice one’s own leg, then one is morally required to make this sacrifice. DE: Wenn in einem Notfall die einzige Möglichkeit, das Leben einer anderen Person zu retten, darin besteht, sein eigenes Bein zu opfern, dann ist dieses Opfer moralisch geboten.
	IB-4	EN: It is just as wrong to fail to help someone as it is to actively harm them yourself. DE: Es ist genauso falsch, jemandem Hilfe zu versagen, wie jemandem selbst aktiv zu schaden.
	IB-5	EN: It is morally wrong to keep money that one doesn’t really need if one can donate it to causes that provide effective help to those who will benefit a great deal. DE: Es ist moralisch falsch, Geld zu behalten, welches man nicht wirklich braucht, statt es Zwecken zu spenden die effektive Hilfe für jene bieten, die daraus großen Nutzen ziehen.
Instrumental Harm	IH-1	EN: It is morally right to harm an innocent person if harming them is a necessary means to helping several other innocent people. DE: Es ist moralisch richtig, einer unschuldigen Person zu schaden, wenn dieser Schaden ein notwendiges Mittel ist, mehreren anderen unschuldigen Personen zu helfen.
	IH-2	EN: If the only way to ensure the overall well-being and happiness of the people is through the use of political oppression for a short, limited period, then political oppression should be used. DE: Wenn die einzige Möglichkeit den Menschen allgemeines Wohlergehen und Glück zu ermöglichen, die Nutzung politischer Unterdrückung für eine kurze, begrenzte Zeit ist, dann sollte politische Unterdrückung genutzt werden.
	IH-3	EN: It is permissible to torture an innocent person if this would be necessary to provide information to prevent a bomb going off that would kill hundreds of people. DE: Wenn es für die Entschärfung einer Bombe, die hunderte Menschen töten würde, notwendig ist, eine unschuldige Person zu foltern, ist dies moralisch zulässig.
	IH-4	EN: Sometimes it is morally necessary for innocent people to die as collateral damage—if more people are saved overall. DE: Manchmal ist es moralisch notwendig, dass unschuldige Menschen als Kollateralschaden sterben – wenn dadurch insgesamt mehr Menschen gerettet werden.

Note: English OUS items reprinted with author permission from Kahane [23]. Pen and paper OUS-DE version can be found in S1 Appendix.

The online study on the SoSciSurvey platform ([89], available at https://www.soscisurvey.de) was set up for the validation of the OUS-DE. Announcements for the online study were distributed by mailing lists on the University of Tübingen’s server as well as printed posters and flyers in the town of Tübingen. The online study ran from May to July 2019. The study was not preregistered.

In the online survey, respondents were first provided with information about the aims of the study, inclusion and exclusion criteria and had to provide written consent via digital button click after reading this information. They were also provided with data protection information for the anonymous survey format. After these procedures, the respondents filled out measurements in the following order: OUS-DE, six moral dilemma scenarios, self-reported agreement with Utilitarianism, Moral Competence Test (MCT, [90]), Moral Foundations Questionnaire (MFQ, [91]), the Saarbrücker Personality Questionnaire (SPF; [92]), the Harm Avoidance Scale (TCI-HA) from the Temperament and Character Inventory (TCI; [93]), and the Action Regulation Emotion Systems Scale (ARES; [94]). In this study only results from OUS-DE, moral dilemma scenarios and self-reported agreement with Utilitarianism will be reported. Original instructions from the questionnaires were included to let the respondents know how to fill out the measurements. Finally, they provided the following demographic information: sex/gender, age, education level, religious affiliation, religiosity, political ideology, mother tongue, existence of diagnosed mental disorders.

After completing the survey, respondents were also provided with an opportunity to win a retail voucher and could enter their e-Mail address into a competition. A voucher competition was used as part of the survey advertisement. Entries from the competition were recorded separately from the survey into a different repository and saved as an independent data file. No identification between survey answers and competition entries was possible as no other information than e-Mail addresses was recorded. The e-Mail addresses were used only for the purpose of voucher competition.

#### Data analysis

Data analyses were performed with IBM SPSS Statistics 28.0.1.1, IBM SPSS Amos 28.0.0 (IBM, Chicago, USA), and R programming language [95] using the *lavaan* package [96].

##### Preliminary checks.

Data distribution for the OUS-DE was first checked for normality using descriptive statistics (skewness and kurtosis). Univariate outliers in OUS-DE responses were identified by Median Absolute Deviations (MAD) test (Tukey’s fence), calculated on the total OUS-DE score, while multivariate outliers were detected by the Mahalanobis Distance in linear regression.

##### Factorial validity.

Confirmatory factor analysis (CFA) was performed to evaluate the factorial structure of the OUS-DE using a maximum likelihood estimation. Both one-factor and two-factor models were tested, with the two latent factors allowed to intercorrelate as in the original OUS validation.

##### Model fit evaluation.

The goodness-of-fit of OUS-DE was assessed by several fit indices, selected based on the original OUS validation report [23] and methodological recommendations [97,98]. Model Chi-square (χ^2^) tests assess the difference between the observed and expected covariance matrixes, where goodness of fit is indicated by a non-significant test result at a p > .05. However, the χ^2^ test is sensitive to sample size, and in our case, it will possibly indicate a poor model fit due to a large sample size [99]. Therefore, we prioritized two incremental fit indices, the non-normed fit index (also called Tucker-Lewis’s index, TLI) and the comparative fit index (CFI) as they adjust for sample size issues (for both: ≥ .90 = acceptable; ≥ .95 = excellent). Additionally, we used the root mean square error of approximation (RMSEA) to measure the discrepancy between the observed covariance matrix and the hypothesized covariance matrix (≤.05 = good;.05–.08 = acceptable; [100]). Lastly, we used the standardized root mean square residual (SRMR) to measure the average of the standardized fitted residuals (≤.08 = acceptable; [100,101]). Parsimony of the measurement models was assessed by Akaike Information Criterion (AIC; [102]) and Bayesian Information Criterion (BIC; [103]). Lower values in both indices show better parsimony in the measurement [97].

##### Reliability.

Internal reliability of the measurement was assessed by split-half method (items split by odd and even positions), with Spearman-Brown and the Guttman split-half coefficients [104,105]. Internal consistency was evaluated using Cronbach’s alpha, where higher values (closer to 1) indicate stronger consistency, accounting for questionnaire length effects [106–108].

##### Construct validity.

Pearson’s two-tailed correlations were calculated between the score of each OUS-DE subscale and the mean score of three corresponding moral scenarios (for more information see Materials of Study 1), as well as between the overall OUS-DE score and self-reported agreement with Utilitarianism.

### Results

#### Data screening and outlier analysis.

Descriptive statistics were first used to assess data normality distribution in overall mean OUS-DE score, scores for each of the two subscales and every item in the scale separately. The mean OUS-DE score was slightly negatively skewed (S = −0.111). IB-DE scores were negatively skewed (S = −0.261), and IH-DE scores were positively skewed (S = 0.292). When each item was considered separately, skewness ranged from negative −0.402 to positive 0.45. Kurtosis statistic showed positive kurtosis for the overall mean OUS-DE score (K = 0.503). Kurtosis negatively but weakly affected IB-DE (K = −0.177) and IH-DE (K = −0.390). When each item was considered separately, kurtosis ranged from −0.999 to −0.399. All reported skewness and kurtosis statistics fall into the acceptable range for large samples [109].

The data was then assessed for univariate and multivariate outliers (see Data analysis section of Study 1). MAD test was performed on the sum of OUS-DE item scores for each participant and the analysis identified one univariate outlier. Mahalanobis Distance in linear regression identified 12 multivariate outliers. These univariate and multivariate outliers were removed from further analyses. After the procedure, final sample consisted of 378 individuals (243 women, M_age_ = 25.37). Mean responses to OUS-DE and its subscales are depicted in Table 3.

**Table 3:** Mean responses to the Oxford Utilitarianism Scale and its subscales.

	Mean score (range)
**OUS-DE**	3.82 (1.00-6.33)
**IB-DE**	4.33 (1.0-7.0)
**IH-DE**	3.19 (1.0-6.75)

#### Psychometric properties of OUS-DE

##### Confirmatory factor analysis (CFA).

CFA was performed on a dataset with 378 cases; no data was missing. Two different models were assessed and compared: 1) a one factor solution and 2) a two fixed factor solution based on the original questionnaire subscales, with covariance drawn between the two factors. Analyses used maximum likelihood estimation (10 iterations). The results are shown in Table 4 and Table 5.

**Table 4:** Results of Confirmatory Factor Analyses (N = 378).

	χ^2^	TLI	CFI	SRMR	RMSEA	AIC	BIC
One-factor default model	564.0, df = 27, p < .001	0.176	0.382	0.186	0.229	12726.737	12797.565
Two-factor default model	92.317, df = 26, p < .001	0.894	0.923	0.0627	0.082	130.317	205.08
Two-factor modified model	56.509, df = 24, p < .001	0.944	0.962	0.0569	0.06	98.509	181.142
IB-DE subscale default model	39.707, df = 5, p < .001	0.806	0.903	0.0532	0.136	59.707	99.056
IB-DE subscale modified model	3.384, df = 3, p = .336	0.996	0.999	0.0155	0.018	27.384	74.603
IH-DE subscale default model	5.602, df = 2, p = .061	0.806	0.903	0.0201	0.069	21.602	53.081
*Recommended value*	*p > .05*	≥*0.90*	≥*0.90*	≤*0.08*	≤*0.07*	*–*	*–*

**Table 5:** Standardized factor loadings for OUS-DE in the CFA (N = 378).

Item	Default one-factor model	Default two-factor model		Modified two-factor model	
IB-1	.568	.591		.573	
IB-2	.697	.690		.610	
IB-3	.664	.637		.544	
IB-4	.397	.407		.444	
IB-5	.573	.604		.610	
IH-1	.212		.724		.723
IH-2	.117		.413		.413
IH-3	.093		.779		.779
IH-4	.085		.849		.850

The default one-factor model clearly showed an inadequate model fit on all fit indices: χ^2^ = 564.0, df = 27, p < .001, TLI = 0.176, CFI = 0.382, SRMR = 0.186, RMSEA = 0.229, AIC = 12726.737, BIC = 12797.565. Similarly, standardized factor loadings indicated that IH-DE items fit poorly with the IB-DE items if one-factor solution is analyzed (see Table 5).

The two-factor model fit was better, though marginally below recommended cut-offs: χ^2^ = 92.317 df = 26, p < .001, TLI = 0.894, CFI = 0.923, RMSEA = 0.082, SRMR = 0.0627. Modification indices suggested that the model could be improved if residual covariances were modelled between questionnaire items IB-2 and IB-3 (MI = 32.453, Standardized EPC = 0.54), as well as IB-1 and IB-5 (MI = 32.111, Standardized EPC = 0.408). Incorporating these adjustments yielded a modified two-factor model with adequate fit: χ^2^ = 56.509 df = 24, p < .001, TLI = 0.944, CFI = 0.962, RMSEA = 0.06, SRMR = 0.0569. Lower AIC and BIC indices of the modified two-factor model also supported this modified model as the preferable solution. Standardized factor loadings (see Table 5) indicated that the two-factor solution for OUS-DE is appropriate (all loadings > .4).

Results of CFA for the two-factor model remained robust when the sample was split according to sex/gender (for analysis descriptions and CFA results in tables see Supporting Information S1 File).

##### Split-half reliability.

Split-half reliability testing was performed on all nine items in the scale. The method of odd-even trials was selected. It estimated a Spearman-Brown Coefficient of 0.71 and a Guttman Split-Half Coefficient of 0.7.

##### Internal consistency.

Internal consistency estimations resulted in Cronbach’s alpha for the whole questionnaire α = 0.671; for IB-DE α = 0.760; and for IH-DE α = 0.716. It is important to note that short questionnaires result in lower alpha values [108], therefore, overall internal consistency could be assumed as acceptable to good.

##### Construct validity.

Construct validity for this measurement was assessed by calculating Pearson’s correlations (two-tailed) between the overall OUS-DE score, IB-DE score, IH-DE score and self-reported Utilitarianism, as well as mean scores of two types of moral dilemmas: a set of dilemmas concerning with greater good and a set concerning with sacrificial harm. As in the original OUS validation study [23], we have assumed that if the OUS-DE truly measures utilitarian inclinations, moral appropriateness ratings in greater good dilemmas will be directly associated with the measures in IB-DE and behavioral results in moral appropriateness ratings in sacrificial harm dilemmas will be directly associated with the measures in IH-DE, and behavioral results in moral decision-making.

The correlation between IB-DE and OUS-DE was strong (r = 0.778, p < .001). The correlation between IH-DE and OUS-DE was also strong (r = 0.662, p < .001).

###### Greater good dilemmas.

**Fig 1:**
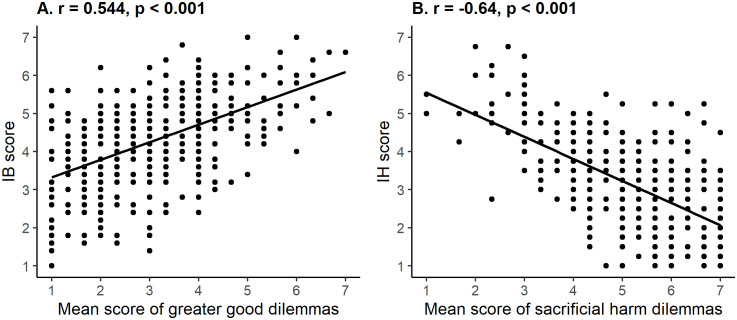
Construct validity analysis. Correlations between responses to IB-DE subscale and greater good dilemmas (A) and between responses to IH-DE subscale and sacrificial harm dilemmas (B).

Mean score of the greater good dilemmas positively correlated with OUS-DE score (r = 0.446, p < .001), IB-DE score (r = 0.544, p < .001) as well as with self-reported Utilitarianism (r = 0.392, p < .001), indicating consistent construct validity. Fig 1 and Table 6 illustrate these correlations.

**Table 6:** Correlations between OUS-DE, its subscales, moral dilemmas, and self-reported Utilitarianism.

	1	2	3	4	5
1. OUS-DE	–				
2. IB-DE	**.78** ^**^	–			
3. IH-DE	**.66** ^**^	.04	–		
4. Sacrificial Harm dilemmas	**−.5** ^**^	**−.13** ^*^	**−.64** ^**^	–	
5. Greater Good dilemmas	**.45** ^**^	**.54** ^**^	.06	**−.11** ^*^	–
6. Self-reported Utilitarianism	**.4** ^**^	**.34** ^**^	**.23** ^**^	**−.2** ^**^	**.39** ^**^

###### Sacrificial harm dilemmas.

Mean score of the sacrificial harm dilemmas, as expected due to scoring difference in these dilemmas with lower scores meaning greater agreement with utilitarian solution, moderately and negatively correlated with OUS-DE score (r = −0.501, p < .001), IH-DE score (r = −0.64, p < .001), as well as with self-reported Utilitarianism (r = −0.204, p < .001), indicating consistent construct validity. Please see Fig 1 and Table 6 for illustration of the correlation.

###### Self-reported utilitarianism.

Self-reported Utilitarianism positively correlated with overall OUS-DE: (r = 0.399, p < .001) as well as IB-DE (r = 0.338, p< .001) and IH-DE (r = 0.231, p < .001). Please see Table 6 for these correlations.

#### Discussion: study 1

In this study, we translated and validated the German version of the Oxford Utilitarianism Scale (OUS-DE). Our results indicate that the OUS-DE is a reliable measure of utilitarian inclinations in young healthy adults, making it an adequate research tool to address utilitarian moral inclinations.

Using a confirmatory factor analysis, a two-factor solution of OUS-DE achieved appropriate model fit. This supports previous findings from the original OUS validation study [23] as well as more recent validation studies of the Turkish [29], Spanish [31], and French [30] OUS versions. A study assessing structural validity for OUS in 15 languages found that a different German OUS translation required multiple modifications to achieve a two-factor solution [32]. In our study, two modifications for the two-factor model were also required to achieve an adequate model fit: in IB-DE subscale, we needed to draw covariances between items IB-2 and IB-3 as well as IB-1 and IB-5. IB-2 (kidney donation) and IB-3 (leg sacrifice) items measure the principle of beneficence, and in particular, one’s agreement with a positive duty to sacrifice one’s body part to save another person’s life in an urgent situation. Due to the similarity of the two items, covariance between these items could have been expected. Covariation between items IB-1 (impartiality) and IB-5 (donation) could also be expected as both items involve referencing of a positive duty to help and care for others. These covariations have not been discovered in the original OUS validation study [23] or other OUS translations. Further studies should investigate possible conceptual overlaps between these items.

It has been recently suggested that the IB subscale should be revised or separated into more fine-grained subscales to represent the general beneficence, self-sacrifice, and action and omission distinction [110]. While covariations between items in this subscale indicated possible conceptual similarity among them, based on our results, we do not recommend a complete revision of the subscale. It is a central commitment of Utilitarianism to consider omissions to help and active harm as commensurable wrongs, as utilitarians consider consequences to be the sole arbiter of wrongness [2,3,22]. Divorcing it from other aspects of Utilitarianism, including impartiality, would lead to a skewed representation of Utilitarianism in the resulting measurement.

Finally, internal consistency of the scale indicated that the translated version of the questionnaire performs comparably well as the original English version and other OUS translations. Construct validity of the translated version was supported by moderate bivariate correlations between OUS-DE and self-reported Utilitarianism, as well as moderate-to-strong correlations between OUS-DE subscales and thematically corresponding moral dilemmas. The latter correlations are comparable with the results of the original OUS validation (see Study 2 in [23]. Moderate-to-strong associations between observations of OUS-DE and moral dilemmas indicate that they are measuring the same construct reliably and might be immune to response style or social desirability effects [111,112].

## Study 2: effects of sociodemographic and personality variables on agreement with OUS-DE

The aim of this study was to investigate the stability of utilitarian inclinations in the general population and the origins of individual differences in self-reported Utilitarianism. To assess the stability of utilitarian inclinations, we have collected responses to OUS-DE and its subscales in two different samples at two different time points: in 2019 and in 2023 when the last protective measures of the Covid-19 pandemic were phased out. Based on previous research [80–82], we assumed that increased exposure to moral situations which require utilitarian solutions would increase agreement with OUS-DE, indicating that external circumstances can have an influence on moral beliefs in a general population.

Furthermore, we investigated individual differences in utilitarian inclinations among individuals due to their sex/gender, age, religiosity, and psychometric measures which conceptually are related to one’s moral dispositions such as moral competence, moral foundations, empathy, harm aversion and approach/avoidance trait. We expected to find a significant sex/gender difference with men reporting higher acceptance of IH-DE than women [50,51,56–59]. We also expected to find a positive relationship between age and IB-DE as well as a negative relationship between age and agreement with IH-DE [31,68]. Furthermore, we anticipated to find a positive relationship between religiosity and IB-DE [23,42]. Finally, positive relationships were expected between agreement with Utilitarianism and moral competence [44], empathy and Harm/Care foundation [15,23,30,35,38], while weak negative relationships were expected between agreement with Utilitarianism and harm aversion as well as behavioral approach trait [39,47–51].

### Materials and methods

#### Sample description.

##### Sample 1.

A sample of N = 378 individuals from Study 1 was included in the analyses in Study 2. (Please see Sample description in Study 1 and Table 1).

##### Sample 2.

Five hundred twenty-six participants completed an online survey from 2022-10-04 to 2023-01-16. The survey consisted of the same measurements as the survey in Sample 1. One hundred seventy eight participants were excluded from further analyses: 32 participants due to a different mother-tongue, nine for failing the two attention checks, 27 for completing the survey below the predetermined minimal time limit (< 20 minutes), five due to non-binary gender identification, 96 due to a self-reported mental disorder diagnosis, three due to an error recording their answers in one of the questionnaire sections. Univariate and multivariate outlier analyses indicated another six participants to be excluded (for more details, please see Data analysis section in Study 1). The final sample consisted of 348 participants (206 women, Mage = 24.61, SD = 6.59). From this sample, 12 participants indicated that they had participated in our first validation survey in 2019, 29 were not sure, and the rest did not participate in the survey in 2019. Please see Table 7 for the sociodemographic characteristics of this sample.

**Table 7:** Sociodemographic Characteristics of Sample 2.

Baseline characteristic	Sample 2 (N = 348)	
	n	%
*Sex/gender*		
Women	206	59,2%
Men	142	40,8%
*Age (years)*		
18-20	108	31,0%
21-25	138	39,7%
26-30	51	14,7%
31-35	19	5,4%
36-40	11	3,2%
41-45	21	6,0%
*Highest education level*		
No completed secondary education	0	0%
Secondary	5	1,4%
General (high school)	104	29,9%
At least one higher education degree	239	68,7%
*Religion*		
Christianity	187	53,7%
Judaism	0	0%
Islam	12	3,4%
Buddhism	0	0%
Agnosticism	43	12,4%
Atheism	95	27,3%
Other	11	3,2%
*Political ideology*		
Marxist/socialist	28	8,0%
Liberal	76	21,8%
Ecologist	79	22,7%
Social democratic	125	35,9%
Conservative	33	9,5%
Nationalistic	7	2,0%

### Materials

#### Oxford Utilitarianism Scale – German translation.

For this study we used the German version of Oxford Utilitarianism Scale, validated in Study 1. The description and the properties of the scale remain exactly as for the OUS described in the Materials section in Study 1.

#### Moral Competence Test, German version.

The Moral Competence Test (MCT; [90,113,114]) is a behavioral test that measures cognitive ability to make consistent judgments about reasons for and acceptability of moral behavior in different moral situations even when the moral behavior opposes subject’s own moral views. The measurement consists of two moral dilemma scenarios and 12 judgment questions for each scenario. The degree of moral competence is measured by the C-score which ranges from 0 to 100, with higher scores indicating higher moral competence. A validated German version of the test was used in this study [113].

#### Moral Foundations Questionnaire, German version.

The Moral Foundations Questionnaire (MFQ-30; [115]) is a self-report measure that assesses the agreement with five foundational moral domains: Harm/Care: approval of harm prevention/relief and disapproval of cruelty and aggression, Fairness/Reciprocity: approval of reciprocal interaction, Ingroup/Loyalty: approval of group solidarity and distrust for outgroup members, Authority/Respect: approval of respect, duty and obedience to authority, and Purity/Sanctity: approval of spirit over body and disapproval of carnal passions [33]. The distinction of the five foundational domains resulted from ethnographic research and evolutionary psychology, and was further developed in the Moral Foundations Theory [116]. Scores range from 0 to 30 in each foundation, with higher scores indicating higher relevance of that foundation to the respondent’s moral intuitions and decision-making. Finally, the MFQ assesses moral progressivism. Progressivism is a social, moral, and political view, which aims to improve society by means of social reform and scientific as well as technological advancement [117]. Subject’s moral progressivism can be measured by subtracting the mean score of Ingroup/Loyalty, Authority/Respect, and Purity/Sanctity foundations from the mean score of Harm/Care and Fairness/Reciprocity foundations. A German version of this questionnaire provided by the official websites of creators and researchers of the MFQ and MFT at www.moralfoundations.org [115] was used in the study.

#### The Saarbrücker Personality Questionnaire.

The Saarbrücker Personality Questionnaire (SPF; [92]) is a German version of the Interpersonality Reactivity Index (IRI; [118]), a common measure of empathy. It consists of four subscales, namely, Perspective Taking, Fantasy, Empathic Concern, and Personal Distress, designed to represent different empathic faculties. The overall empathy score is calculated as a sum of the scores of the three subscales Perspective Taking, Fantasy, and Empathic Concern. The Personal Distress subscale is excluded from the empathy score as it measures emotion regulation in empathic situations. The Personal Distress score (negatively scored) can be subtracted from the overall empathy score to calculate the overall score of the questionnaire.

#### Harm Avoidance Scale from Temperament and Character Inventory, German version.

The Harm Avoidance Scale (TCI-HA) from the Temperament and Character Inventory (TCI; [93]) consists of 35 items and measures temperament traits related to harm-avoidant and anxiety-expressing behaviors, like action inhibition, avoidance of punishment, novel stimuli, or non-rewarding actions. Four subscales constitute the TCI-HA: anticipatory worry vs uninhibited optimism (HA1), fear of uncertainty vs confidence (HA2), shyness with strangers vs gregariousness (HA3), and fatigability and asthenia vs vigor (HA4). Overall, the TCI-HA score is calculated as a sum of the four subscales. Higher scores in the whole scale indicate higher harm aversion and higher anticipatory worry, higher fear of uncertainty, more pronounced shyness with strangers, as well as lower mental and physical energy.

#### Action Regulation Emotion Systems Scale.

The Action Regulation Emotion Systems Scale (ARES; 94) is a measure developed in Germany to measure the Behavioral Inhibition System (BIS) and the Behavioral Approach System (BAS), and is based on the BIS/BAS-Scale by Carver and White [119]. In ARES, the BIS scale assesses the subject’s proneness to inhibit behavior that might lead to negative outcomes or feelings. Similarly, BAS scale measures proneness to engage in behaviors that bring about positive (rewarding) outcomes and feelings. Additionally, ARES assesses proneness to anger as a reaction to failure of a goal-directed action in a separate subscale [94]. The long version of the questionnaire was implemented in this study (58 items), which consists of three scales: 1) BIS, which is further split into two subscales BIS I “Anxiety” and BIS II “Frustration”, 2) Anger, and 3) BAS, which is divided into two subscales BAS I “Drive” and BAS II “Gratification”.

### Procedure.

The Ethics commission of the Medical Faculty at the University of Tübingen approved the online survey (“Validation of German translation of Oxford Utilitarianism Scale”, project number 839/2018BO2).

The online study was sept up on the SoSciSurvey platform (89, available at https://www.soscisurvey.de) and was identical to Study 1. Please see description of the study’s procedure in Study 1. Only an additional final question was included in this study, asking the respondents whether they believe that they have already participated in the identical study in 2019. The online study ran from January to April 2022. The study was not preregistered.

### Data analysis.

Data analyses were performed with IBM SPSS Statistics 28.0.1.1 and R programming language [95].

#### Preliminary checks.

The distribution of OUS-DE scores was examined for normality using descriptive statistics, including assessments of skewness and kurtosis. Univariate and multivariate outlier analysis was performed using the same procedures as in Study 1 to ensure consistency.

#### Multivariate group comparisons.

To investigate the joint effects of sex/gender and sample (measurement time) on the dependent variables (i.e., OUS-DE, IB-DE, IH-DE) we conducted a Multivariate Analysis of Variance (MANOVA). This approach was chosen as it accounts for conceptual and statistical relationships among dependent variables while controlling for Type I error inflation. In this study, sex/gender and measurement time (sample) served as fixed factors, with the three OUS-DE measures as dependent variables. Prior to analysis, assumptions of normality, homogeneity of variance-covariance matrices, and absence of multicollinearity were checked. In cases where assumptions for parametric testing were violated, robust test statistics were used (e.g., Pillai’s Trace for MANOVA; Welch’s ANOVA for univariate follow-ups).”

#### Linear associations.

Pearson’s two-tailed correlations were computed to examine associations between OUS-DE scores and personality measures, age, and religiosity. The strength of correlations was interpreted as very strong: r ≥ .8; strong:.8 > r ≥ .6; moderate:.60 > r ≥ .4; weak:.4 > r ≥ .2; very weak: r < .2 [120]. The Benjamini-Hochberg procedure [121] was applied to control the false discovery rate (FDR) as correction for multiple comparisons within logically defined correlation families. Applied to meaningful hypothesis groups, this procedure provides a sensitive correction, while reliably controlling false discoveries and family-wise error. It also accommodates dependency between tests and preserves statistical power when a large number of tests are performed [122].

## Results

### Associations between OUS-DE and sociodemographic measures

#### Sex/gender and sample effects.

A two-way MANOVA was conducted to examine the effects of sex/gender and sample on subscales of OUS-DE. The analysis revealed significant multivariate main effects of sex/gender (Pillai’s Trace = 0.026, F(2, 721) = 9.5, p < .001), and sample (Pillai’s Trace = 0.01, F(2, 721) = 3.7, p = .025), but no significant interaction between the two factors (Pillai’s Trace = 0.002, F(2, 721) = 0.73, p = .48). This indicates that sex/gender and sample each independently contributed to differences in the dependent variables. For group means of IB-DE, IH-DE, and each of the IB-DE items by sample and sex/gender, please see Table 8.

**Table 8:** Mean scores of IB-DE, IH-DE, and IB-DE items in Sample 1 and Sample 2 as well as women and men across the samples.

	Group means by sample		Group means by sex/gender	
	Sample 1 (N = 378)	Sample 2 (N = 348)	Women (N = 449)	Men (N = 277)
IB-DE	4.33 (1.17)	4.09 (1.06)	4.35 (1.04)	4.07 (1.22)
IH-DE	3.19 (1.23)	3.31 (1.21)	3.2 (1.13)	3.31 (1.35)
IB-1	4.27 (1.75)	4.22 (1.77)	4.4 (1.64)	4.0 (1.91)
IB-2	4.35 (1.7)	4.25 (1.7)	4.39 (1.64)	4.16 (1.78)
IB-3	4.38 (1.61)	4.31 (1.63)	4.46 (1.53)	4.16 (1.74)
IB-4	4.65 (1.76)	3.83 (1.81)	4.46 (1.74)	3.93 (1.93)
IB-5	3.19 (1.23)	3.31 (1.21)	4.07 (1.59)	3.69 (1.74)

Given the significant multivariate main effects, follow-up univariate ANOVAs were conducted separately for sex/gender and sample. Interaction effects were not examined further at the univariate level because they were non-significant in the MANOVA.

#### IB-DE.

**Fig 2:**
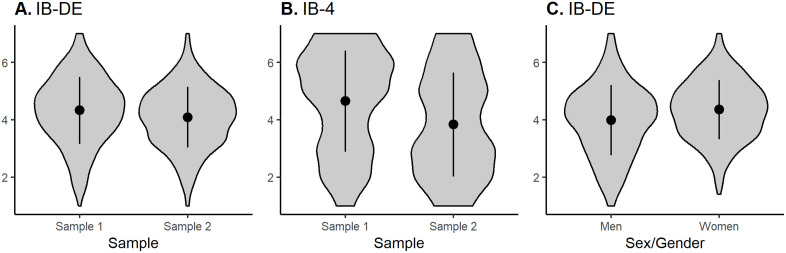
Violin plots showing the distribution of IB-DE and IB-4 across samples and sexes. A and B display group differences between Sample 1 and Sample 2 in IB-DE and IB-4 (Y axis depicts scores of A. IB-DE and B. IB-4), respectively, while C illustrates sex/gender differences in IB-DE (Y axis depicts scores of IB-DE). Each violin represents the probability density of the data, with the point range indicating the mean ± standard deviation.

For sample, a one-way ANOVA also revealed a significant effect, F(1, 724) = 8.23, p = .004, η² = 0.011, with higher values in Sample 1 (please see Fig 2A). For sex/gender, the Welch’s ANOVA revealed a significant difference in IB-DE mean scores, F(1, 513) = 17.13, p < .001, η² = 0.025, with higher values in women (please see Fig 2C).

#### IH-DE.

For sample, a one-way ANOVA yielded no significant effect, F(1, 724) = 1.69, p = .194, η² = 0.002. Similar for sex/gender, the Welch’s ANOVA showed no significant difference in IH-DE mean scores, F(1, 509) = 1.13, p = .289, η² = 0.002.

#### IB-DE items.

To analyze which IB-DE items were influenced by multivariate effects of sex/gender and sample, another two-way MANOVA was performed. As expected, multivariate main effects were significant for sex/gender (Wilks’ Lambda = 0.954, F(5, 718) = 7.0, p < .001) and sample (Wilks’ Lambda = 0.971, F(5, 718) = 4.31, p < .001) but not their interaction (Wilks’ Lambda = 0.999, F(5, 718) = 0.14, p = .983), again indicating independent contribution of sex/gender and sample on dependent variables.

For sample, separate one-way ANOVAs with Bonferroni correction of α = 0.01 showed one significant effect of sample on the IB-4 item, F(1, 724) = 38.01, p < .001, η² = 0.05 (all other p’s > .99), with higher values in Sample 1 (please see Fig 2B). For sex/gender, separate Welch’s ANOVAs with Bonferroni correction of α = 0.01 indicated significant sex/gender effects for three items – IB1, F(1, 519.5) = 8.21, p = .02, η² = 0.012, IB4, F(1, 539.5) = 11.94, p < .001, η² = 0.02, and IB5, F(1, 540.6) = 8.47, p = .02, η² = 0.012 (all other p’s > 0.1), with higher values in women.

#### Age.

Weak negative correlations between age and OUS-DE (r_S1_ = −0.152, p_S1_ = .003; r_S2_ = −0.15, p_S2_ = .005) and IH-DE (r_S1_ = −0.15, p_S1_ = .004; r_S2_ = −0.12, p_S2_ = .026) subscale were found in both samples. No correlation between age and IB-DE subscale was found in both samples (all p’s > .065).

#### Religiosity.

A weak positive correlation between frequency of one’s religious practice and IB-DE was found in Sample 1 only (r_S1_ = 0.124, p_S1_ = .016). No correlations between religious practice and OUS-DE or IH-DE were found in both samples (all p’s > .079), while religious practice and IB-DE did not correlate in Sample 2 (p = .19).

### Associations between OUS-DE and psychometric measures

Correlations with psychometric measures were assessed by correlation analyses (Pearson’s r, two-tailed significance test) in Sample 1 (N = 378) and Sample 2 (N = 348). Correction for multiple comparisons was applied to logical test families according to Benjamini-Hochberg procedure. Results of associations between OUS-DE and its subscales with personality measures are depicted in Table 9 for Sample 1 and Table 10 for Sample 2.

**Table 9:** Correlations between OUS-DE, its subscales and personality measurements in Sample 1.

	1	2	3	4	5	6	7	8	9	10	11	12	13	14	15	16	17	18	19	20	21	22	23	24	25	26	27
1. OUS-DE	–																										
2. IB-DE	0.78**	–																									
3. IH-DE	0.66**	0.04	–																								
4. Moral competence	0.09	0.10	0.02	–																							
5. Harm/Care foundation (MFQ-30)	0.21**	0.39**	−0.14*	−0.06	–																						
6. Fairness/Reciprocity foundation (MFQ-30)	0.31**	0.41**	0.00	0.00	0.44**	–																					
7. Ingroup/Loyalty foundation (MFQ-30)	0.05	−0.02	0.11	−0.03	0.21**	0.00	–																				
8. Authority/Respect foundation (MFQ-30)	−0.02	−0.14*	0.13*	−0.09	0.08	−0.13*	0.52**	–																			
9. Purity/Sanctity foundation (MFQ-30)	0.03	0.04	0.00	−0.12	0.35**	0.01	0.41**	0.56**	–																		
10. Progressivism (MFQ-30)	0.15*	0.33**	−0.16**	0.05	0.36**	0.59**	−0.49**	−0.70**	−0.56**	–																	
11. Fantasy (SPF)	0.02	0.02	0.01	−0.07	0.18**	0.17**	0.02	0.06	0.17**	0.04	–																
12. Perspective taking (SPF)	0.08	0.20**	−0.11*	−0.03	0.26**	0.20**	−0.06	−0.09	−0.05	0.25**	0.16**	–															
13. Empathic concern (SPF)	0.18**	0.30**	−0.07	−0.01	0.46**	0.30**	0.10	−0.02	0.21**	0.20**	0.48**	0.39**	–														
14. Personal distress (SPF)	0.17**	0.12*	0.12*	−0.01	0.19**	0.15**	0.01	0.08	0.17**	0.02	0.23**	−0.02	0.23**	–													
15. Overall empathy (SPF)	0.12*	0.21**	−0.07	−0.05	0.39**	0.29**	0.03	−0.01	0.16**	0.20**	0.78**	0.64**	0.83**	0.21**	–												
16. Overall SPF score	0.04	0.15**	−0.12*	−0.05	0.30**	0.22**	0.02	−0.05	0.08	0.19**	0.66*	0.64**	0.71**	−0.26**	0.89**	–											
17. Anticipatory worry vs uninhibited optimism (TCI HA1)	0.02	−0.02	0.06	−0.02	0.05	0.00	−0.04	0.04	0.07	0.00	0.15	−0.14	0.04	0.45**	0.04	−0.17**	–										
18. Fear of uncertainty vs confidence (TCI HA2)	0.03	−0.01	0.06	0.01	0.17*	0.13	0.00	0.09	0.18*	0.00	0.11	−0.01	0.10	0.52**	0.09	−0.15*	0.40**	–									
19. Shyness with strangers vs gregariousness (TCI HA3)	−0.02	−0.04	0.02	0.01	0.00	−0.01	−0.12	−0.01	0.05	0.01	0.01	−0.10	−0.04	0.43**	−0.05	−0.25**	0.38**	0.44**	–								
20. Fatigability and asthenia vs vigor (TCI HA4)	−0.04	−0.06	0.00	−0.02	0.12	0.05	−0.04	0.01	0.03	0.05	0.14*	−0.04	0.10	0.34**	0.10	−0.06	0.42**	0.35**	0.31**	–							
21. Overall harm avoidance (TCI HA)	0.00	−0.04	0.05	−0.01	0.11	0.05	−0.06	0.05	0.11	0.02	0.14*	−0.10	0.07	0.59**	0.06	−0.21**	0.80**	0.72**	0.69**	0.71**	–						
22. Anxiety (ARES BIS I)	0.04	0.01	0.06	−0.01	0.19**	0.11*	0.00	0.08	0.10	0.05	0.25**	−0.04	0.20**	0.58**	0.20**	−0.07	0.57**	0.48**	0.46**	0.44**	0.67**	–					
23. Frustration (ARES BIS II)	0.06	0.00	0.09	0.01	0.20**	0.13*	0.04	0.12*	0.15**	0.02	0.25**	−0.12*	0.18**	0.45**	0.16**	−0.05	0.56**	0.34**	0.35**	0.39**	0.57**	0.75**	**–**				
24. Overall Behavioral Inhibition System (ARES BIS)	0.06	0.01	0.08	0.00	0.21**	0.13*	0.02	0.11	0.14*	0.04	0.26**	−0.09	0.20**	0.54**	0.19**	−0.07	0.60**	0.43**	0.43**	0.44**	0.66**	0.92**	0.95**	**–**			
25. Anger (ARES)	0.00	−0.07	0.08	0.00	0.08	0.05	0.12*	0.2**	0.18**	−0.11*	0.22**	−0.16**	0.06	0.31**	0.07	−0.07	0.39**	0.20**	0.16**	0.28**	0.36**	0.50**	0.69**	0.64**	–		
26. Drive (ARES BAS I)	0.07	0.11	−0.02	−0.07	0.20**	0.15**	0.14*	0.12*	0.16**	−0.01	0.20**	0.18**	0.34**	0.00	0.32**	0.31**	−0.28**	−0.04	−0.28**	−0.20**	−0.28**	−0.12*	−0.07	−0.10	0.01	–	
27. Gratification (ARES BAS II)	0.07	0.10	−0.02	−0.12	0.19**	0.13*	0.12*	0.14*	0.16**	−0.01	0.27**	0.20**	0.31**	−0.08	0.35**	0.38**	−0.16**	−0.08	−0.26**	−0.21**	−0.24**	−0.04	0.08	0.02	0.19**	0.65**	–
28. Overall Behavioral Activation System (ARES)	0.07	0.11	−0.02	−0.10	0.22**	0.16**	0.15*	0.14*	0.18**	−0.01	0.25**	0.21**	0.36**	−0.04	0.36**	0.38**	−0.25**	−0.07	−0.30**	−0.23**	−0.29**	−0.09	0.00	−0.05	0.10	0.92**	0.90**

**. Correlation is significant at the 0.01 level (2-tailed, Benjamini-Hochberg correction for multiple comparisons applied).

*. Correlation is significant at the 0.05 level (2-tailed, Benjamini-Hochberg correction for multiple comparisons applied).

**Table 10:** Correlations between OUS-DE, its subscales and personality measurements in Sample 2.

	1	2	3	4	5	6	7	8	9	10	11	12	13	14	15	16	17	18	19	20	21	22	23	24	25	26	27
1. OUS-DE	–																										
2. IB-DE	0.76**	–																									
3. IH-DE	0.70**	0.07	–																								
4. Moral competence	−0.05	−0.09	0.03	–																							
5. Harm/Care foundation (MFQ-30)	0.16**	0.35**	−0.13*	−0.11	–																						
6. Fairness/Reciprocity foundation (MFQ-30)	0.15*	0.27**	−0.07	−0.02	0.41**	–																					
7. Ingroup/Loyalty foundation (MFQ-30)	−0.08	−0.13*	0.02	−0.03	0.13*	−0.03	–																				
8. Authority/Respect foundation (MFQ-30)	−0.15*	−0.31**	0.11	0.00	−0.02	−0.22**	0.59**	–																			
9. Purity/Sanctity foundation (MFQ-30)	−0.13*	−0.15*	−0.04	−0.08	0.22**	−0.02	0.52**	0.60**	–																		
10. Progressivism (MFQ-30)	0.23**	0.42**	−0.10	−0.01	0.45**	0.59**	−0.59**	−0.77**	−0.59**	–																	
11. Fantasy (SPF)	0.10	0.14*	0.00	0.05	0.23**	0.13*	0.03	0.00	0.05	0.11	–																
12. Perspective taking (SPF)	0.05	0.13*	−0.07	−0.02	0.27**	0.15*	0.00	0.00	−0.02	0.17**	0.31**	–															
13. Empathic concern (SPF)	0.19**	0.33**	−0.08	−0.03	0.50**	0.27**	0.10	−0.08	0.11	0.26**	0.47**	0.35**	–														
14. Personal distress (SPF)	0.01	0.03	−0.02	0.00	0.14*	0.12	0.00	0.07	0.05	0.06	0.14**	−0.01	0.21**	–													
15. Overall empathy (SPF)	0.15*	0.26**	−0.06	0.00	0.43**	0.24**	0.06	−0.03	0.06	0.23**	0.80**	0.71**	0.79**	0.16**	–												
16. Overall SPF score	0.14*	0.23**	−0.05	0.00	0.35**	0.17**	0.06	−0.07	0.03	0.20**	0.71**	0.69**	0.67**	−0.30**	0.90**	–											
17. Anticipatory worry vs uninhibited optimism (TCI HA1)	0.05	−0.03	0.11	0.02	−0.02	0.03	−0.04	0.02	0.04	−0.01	0.09	−0.08	0.09	0.42**	0.05	−0.14*	–										
18. Fear of uncertainty vs confidence (TCI HA2)	0.02	−0.03	0.06	−0.02	0.08	0.06	−0.10	0.04	−0.02	0.08	0.09	−0.02	0.11	0.46**	0.08	−0.13*	0.41**	–									
19. Shyness with strangers vs gregariousness (TCI HA3)	0.06	0.03	0.06	−0.04	0.04	0.07	0.01	0.07	0.07	−0.01	−0.01	0.00	−0.02	0.40**	−0.01	−0.20**	0.39**	0.39**	–								
20. Fatigability and asthenia vs vigor (TCI HA4)	0.06	0.02	0.07	0.06	0.14	0.11	0.03	0.03	−0.01	0.08	0.20**	0.02	0.22**	0.34**	0.20**	0.04	0.33**	0.39**	0.30**	–							
21. Overall harm avoidance (TCI HA)	0.06	−0.01	0.11	0.01	0.08	0.09	−0.04	0.05	0.03	0.05	0.13*	−0.03	0.14*	0.56**	0.11	−0.14*	0.78**	0.73**	0.69**	0.69**	–						
22. Anxiety (ARES BIS I)	0.03	0.03	0.01	0.03	0.22**	0.09	0.03	0.10	0.10	0.05	0.21**	0.07	0.30**	0.59**	0.26**	−0.01	0.57**	0.55**	0.48**	0.46**	0.71**	–					
23. Frustration (ARES BIS II)	0.07	0.02	0.09	0.04	0.12	0.09	0.07	0.12	0.13*	−0.02	0.20**	−0.05	0.25**	0.45**	0.18**	−0.03	0.54**	0.43**	0.35**	0.40**	0.60**	0.75**	**–**				
24. Overall Behavioral Inhibition System (ARES BIS)	0.06	0.03	0.06	0.04	0.18**	0.09	0.05	0.12	0.12	0.01	0.22**	0.00	0.29**	0.55**	0.23**	−0.02	0.59**	0.52**	0.43**	0.46**	0.70**	0.92**	0.95**	**–**			
25. Anger (ARES)	−0.06	−0.15	0.08	−0.05	0.06	0.11	0.18**	0.26**	0.28**	−0.17**	0.05	−0.21**	0.05	0.30**	−0.04	−0.17**	0.34**	0.26**	0.24**	0.22**	0.37**	0.45**	0.62**	0.58**	–		
26. Drive (ARES BAS I)	−0.04	0.08	−0.15	0.01	0.27**	0.11	0.10	0.05	0.07	0.08	0.23**	0.24**	0.38**	0.02	0.37**	0.35**	−0.26**	−0.03	−0.26**	−0.05	−0.21**	0.00	0.04	0.03	−0.01	–	
27. Gratification (ARES BAS II)	0.00	0.04	−0.05	0.01	0.21**	0.14*	0.13*	0.05	0.15*	0.03	0.19**	0.14*	0.27**	0.06	0.26**	0.22**	−0.06	−0.05	−0.16**	−0.12*	−0.13*	0.08	0.23**	0.17**	0.22**	0.61**	–
28. Overall Behavioral Activation System (ARES)	−0.03	0.07	−0.12	0.01	0.27**	0.14*	0.13*	0.06	0.12	0.06	0.23**	0.21**	0.37**	0.05	0.35**	0.32**	−0.18**	−0.05	−0.24**	−0.09	−0.19**	0.04	0.15**	0.11	0.11	0.90**	0.89**

**. Correlation is significant at the 0.01 level (2-tailed, Benjamini-Hochberg correction for multiple comparisons applied).

*. Correlation is significant at the 0.05 level (2-tailed, Benjamini-Hochberg correction for multiple comparisons applied).

#### Moral competence.

Both in Sample 1 and Sample 2, no correlations between moral competence score and individual OUS-DE scores or scores of the IB-DE and IH-DE reached significance (all p’s > .09).

#### Moral foundations.

Individual OUS-DE scores positively and weakly correlated with Harm/Care (r_S1_ = 0.21, p_S1_ < .001, r_S2 _= 0.16, p_S2 _< .001), Fairness/Reciprocity (r_S1_ = 0.31, p_S1_ < .001, r_S2_ = 0.15, p_S2_ = .01), and Progressivism (r_S1_ = 0.15, p_S1_ = .01, r_S2 _= 0.23, p_S2 _< .001) in both samples. In Sample 2, OUS-DE scores also negatively and weakly correlated with Authority/Respect (r_S2_ = −0.14, p_S2_ = 0.01) and Purity/Sanctity (r_S2_ = −0.13, p_S2_ = 0.025). No correlation was found between the mean OUS-DE score and Ingroup/Loyalty in both samples (p > .1).

IB-DE scores positively correlated with Harm/Care (r_S1_ = 0.39, p_S1_ < .001, r_S2_ = 34, pS2 < .001) and Fairness/Reciprocity (r_S1_ = 0.40, p_S1_ < .001, r_S2_ = 0.27, p_S2_ < .001) as well as Progressivism (r_S1_ = 0.33, p_S1_ < .001, r_S2 _= 0.42, p_S2_ < .001) in both samples. It negatively and weakly correlated with Authority/Respect (r_S1_ = −0.14, p_S1_ = 0.01, r_S2_ = −.31, p_S2_ < .001) in both samples, and with Ingroup/Loyalty (r_S2_ = −.13, p_S2_ = .025) as well as Purity/Sanctity (r_S2_ = −.15, p_S2_ = .01) in Sample 2.

IH-DE negatively and weakly correlated with Harm/Care (r_S1_ = −0.14, p_S1_ = 0.01, r_S2_ = −0.13, p_S2_ = .026) in both samples and Progressivism (r_S1_ = −0.16, p_S1_ < .001) in Sample 1. It was also positively associated with Authority/Respect (r_S1_ = 0.13, p_S1_ = 0.02) in Sample 1. Correlations between IH-DE and Fairness/Reciprocity, Ingroup/Loyalty, and Purity/Sanctity did not reach statistical significance in both samples, as well as Authority/Respect and Progressivism in Sample 2 (all p’s > 0.05).

#### Empathy.

Individual OUS-DE scores positively but weakly correlated with the Empathic Concern subscale (r_S1_ = 0.18, p_S1_ = .002, r_S2_ = 0.19, p_S2_ = .002) and the overall Empathy score (r_S1_ = 0.12, p_S1_ = 0.04, r_S2_ = 0.15, p_S2_ = .02) in both samples. In Sample 1 they also positively and weakly correlated with the Personal Distress subscale (r_S1_ = 0.17, p_S1_ = .004). In Sample 2 they positively and weakly correlated with the SPF total score (r_S2_ = 0.14, p_S2_ = 0.03).

IB-DE positively correlated with Perspective Taking (r_S1_ = 0.20, p_S1_ < .001, r_S2_ = 0.13, p_S2_ = .04), Empathic concern (r_S1_ = 0.30, p_S1_ < .001, r_S2_ = 0.33, p_S2_ < .001), Empathy score (r_S1_ = 0.21, p_S1_ < .001, r_S2_ = 0.26, p_S2_ < .001), and SPF total score (r_S1_ = 0.15, p_S1_ = .008, r_S2_ = 0.23, p_S2_ < .001) in both samples. In Sample 1 it also positively correlated with Personal Distress (r_S1_ = 0.12, p_S1_ = 0.04), while in Sample 2 it correlated with Fantasy (r_S2_ = 0.14, p_S2_ = .02).

IH-DE negatively correlated with Perspective Taking (r_S1_ = −0.11, p_S1_ = 0.046) as well as the SPF total score (r_S1_ = −0.12, p_S1_ = 0.04) and was associated positively with Personal Distress (r_S1_ = 0.12, p_S1_ = 0.04) in Sample 1. In Sample 2, no significant correlations were found (all p’s > 0.3).

#### Harm avoidance.

The individual OUS-DE and IB-DE scores showed no correlations with TCI-HA or its subscales in both samples (all p’s > .89).

#### Behavioral inhibition and activation.

The individual OUS-DE, IH-DE, and IB-DE scores did not correlate with ARES scores in both samples (all p’s > .05).

### Discussion: Study 2

#### Differences in utilitarian inclinations before and after the Covid-19 pandemic.

During the Covid-19 pandemic individuals worldwide were confronted with an unusually high number of moral dilemmas involving both instrumental harm and impartial beneficence [71–73]. Therefore, we hypothesized that prolonged exposure to such situations would increase agreement with utilitarian principles. To examine this, we compared scores of the OUS-DE and its subscales in two independent samples collected before and after the pandemic.

We expected to see a change in IH-DE ratings, with higher acceptance of instrumental harm after the pandemic, as the pandemic uniquely exposed society to sacrificial dilemmas [78]. Surprisingly, no differences were found for IH-DE or combined OUS-DE scores, suggesting that acceptance of instrumental harm – and general utilitarian outlook – remained stable. This aligns with prior research showing either no change [79,123] or mixed effects [28,124] in sacrificial moral dilemmas over time, although some studies report increases in acceptance of utilitarian solutions in pandemic-specific triage contexts [25,123–125]. Taken together, this supports the idea that general attitudes toward instrumental harm are temporally stable, while their application and more nuanced formulations may shift depending on situational salience and personal proximity to the dilemma.

In contrast, IB-DE ratings significantly decreased in the post-pandemic sample, driven primarily by lower agreement with IB-4 (“failing to help”). This change may reflect “moral fatigue”: during the pandemic, individuals – regardless of personal risk – were repeatedly urged to take burdensome protective actions for the benefit of anonymous others [126–128]. While such appeals were effective early on, prolonged exposure to invisible or diffuse harms may have eroded motivation, leading to reduced endorsement of impartial beneficence [129–131]. This hypothesis fits well as many countries, including Germany, saw significant pushback against regulations, both in the form of nonviolent protest and violent escalation (for example, see news reports [132,133]).

An alternative, more speculative explanation relates to the timing of data collection in Sample 2 (from October 2022 to January 2023), during Russia’s invasion of Ukraine. Media coverage during this period heavily featured debates over the extent on how much financial, humanitarian, and military aid the German government should provide to the Ukraine [134]. Public opinion polls in Germany have shown a change in attitudes towards military actions and beneficence in the general population, indicating that after the invasion of Ukraine, the German population more strongly condemned offensive military actions, yet supported diplomatic pressure and military aid, and strongly supported carrying additional personal costs due to sanctions to the aggressor, as well as engaged personally in civil helping behaviors [135,136]. Under these conditions, acts of omission (failure to help) may have been perceived differently, as societal focus shifted toward collective, high-cost assistance and visible political action.

Overall, the findings suggest that while instrumental harm attitudes remained robust to major societal disruptions, impartial beneficence, particularly regarding obligations to help strangers, may be more susceptible to contextual and temporal influences.

### Sex/gender differences in utilitarian inclinations.

Our results indicate a significant sex/gender difference in the mean rating of the IB-DE subscale. On average, women agreed with the IB-DE subscale more than men. Subsequent analyses indicated that the sex/gender difference in IB-DE is driven by independent sex/gender effects on items IB-1 (impartiality), IB-4 (failing to help), and IB-5 (donation). Sex/gender differences in charitable giving and altruism, concepts that are close to beneficence, have been observed consistently in behavioral paradigms (e.g., [60,61] for meta-analysis). Similar results were recently obtained in a German adult sample with women scoring higher in altruistic, emotional, and compliant prosocial behavior than men [137].

Gender roles and gender identification, rather than biological sex, have been suggested to underly these differences. Research shows that women and feminine-identifying individuals tend to view moral dilemmas as more important than men and masculine-identifying individuals [138], suggesting a greater motivation to engage in prosocial behavior. Moreover, gender role orientation has been found to relate more strongly to care-based moral reasoning than biological sex [139]. Women’s generally higher empathic abilities are often linked to their greater prosocial tendencies [138,140,141]. In addition, previous research indicates that women place greater emphasis on fairness, a concept closely related to impartiality, than men, particularly in WEIRD societies [53]. Previous literature has also identified a social desirability bias in ethical responding, with women more likely than men to answer in ways deemed socially acceptable, potentially inflating observed sex/gender differences [142]. In this study, we did not correct for this bias. Taken together, sex/gender differences in agreement with IB-DE may reflect a combination of differences in other moral dispositions.

Contrary to our expectations and previous behavioral findings in sacrificial dilemmas, where men were more accepting of direct sacrificial harm [50,51,56–58,140] and self-serving harm [143], we found no sex/gender differences on IH-DE subscale. One possible explanation might be that women and men do not differ in sacrificial harm-related moral beliefs per se, but diverge in behavior when applying these beliefs, influenced by factors such as gender role expectations in care-based morality [144,145] or the experimental framing of harm-related dilemmas [51].

### Relationships between OUS-DE and other psychometric measures.

In our study we examined the psychometric properties of the OUS-DE, with a particular focus on the associations between personality, character, and utilitarian inclinations.

#### Moral foundations.

Across both samples, we consistently found that agreement with OUS-DE and IB-DE was positively associated with Harm/Care, whereas IH-DE was negatively associated with Harm/Care. This suggests that IB-DE scores were driving the correlation between overall OUS-DE scores and the Harm/Care foundation. In line with our findings, Amormino [35] reported that extraordinary altruists scoring high on IB also show a greater concern for the Harm/Care foundation. We also found associations between IB-DE and the Fairness/Reciprocity foundation in both samples. This is consistent with the view that impartial treatment of individuals is typically seen as a core component of fairness and justice, as argued by many philosophers (e.g., [3,146–149]). Finally, IB-DE was negatively associated with Authority/Respect in both samples. Previous research shows that individuals concerned with the Authority/Respect foundation prefer benefiting their in-group members and have a low concern for impartiality or beneficence towards out-group members [150–152]. Together, these results show that the OUS-DE subscales are distinctly — and in the case of Harm/Care, oppositely — related to broader moral concerns, highlighting the importance of examining subscale-level relationships rather than relying solely on overall Utilitarianism scores.

#### Empathy.

In this study, we found that in both samples OUS-DE scores were positively associated with perspective taking, empathic concern and overall empathy. The association between OUS-DE and empathic concern replicates the findings of the original study [23] and the French validation (OUS-Fr, 30) but contrasts with the Turkish validation [29], despite all three using the same empathy questionnaire. This pattern suggests that correlations between utilitarian inclinations and empathy might be shaped by cultural differences, particularly differences between WEIRD and non-Western contexts.

Across both samples, IH-DE showed no correlation with empathic concern. In Sample 1, IH-DE was negatively correlated with perspective taking and consequently with the overall scale score. This finding is consistent with prior research that empathy, both as a personality trait and as a cognitive ability [153] is generally positively associated with prosocial moral beliefs [154–156] but negatively associated with endorsement of instrumental harm [157,158]. However, other studies indicate that the relationship between empathy, prosocial tendencies, and acceptance of sacrificial harm is dependent on gender or gender role orientations [138,141,159].

Similarly to the divergent associations observed between moral foundations and the OUS-DE subscales, we also found an opposite association between the subscales and perspective taking. Taken together, these results support the notion that the two OUS-DE subscales capture distinct, and in some respect opposing psychological dispositions, consistent with the two-dimensional model of utilitarian psychology [15,23]. However, future research is needed to better characterize individuals who endorse both tenets of Utilitarianism simultaneously. Such a profile may reflect a relatively rare moral phenotype, defined by a distinctive constellation of cognitive and emotional traits. In the context of perspective taking, these individuals may uniquely combine a strong ability to adopt others’ viewpoints with a willingness to endorse instrumental harm when it serves impartial benefit.

#### Moral competence, harm avoidance, and approach/avoidance.

In this study, we found no significant correlations in both samples between Utilitarianism, moral competence, harm avoidance, or approach/avoidance traits. While we cannot conclude from our results any causal explanations on whether highly utilitarian individuals base their moral inclinations on more cognitive approaches, our analysis showed that in both samples, there was a mix of individuals scoring low, medium, and high on MCT regardless of their agreement with Utilitarianism. Furthermore, it is important to note that in our study, avoidance of self-harm as opposed to avoidance of harming others was measured. It is possible that these two types of harm avoidance share little overlap [160]. While there is no validated self-report measure of avoidance of harming others, future research should investigate this aspect and its relation to Utilitarianism.

Previous behavioral studies have linked moral competence, harm avoidance, and approach/avoidance traits to Utilitarianism [47,161,162]. However, while these traits relate to utilitarian behavior through cognitive and affective mechanisms, they might not be tied to moral inclinations per se. It has been suggested that moral beliefs do not directly translate to moral behavior [83] but instead are applied flexibly depending on moral context, state of sobriety, stress level and other external factors. These factors influence mental states, cognitive control, emotion processing, value coding, moral norm internalization and their interactions [163–170]. Therefore, our results add to the growing body of evidence suggesting that moral inclinations do not necessarily translate directly into moral behavior.

## Study 3: Relationships between utilitarian inclinations and real-life moral judgment and behavior

Our results in Study 2, along with previous research, indicate that moral inclinations are not directly associated with moral behavior [83]. We aimed to investigate whether utilitarian inclinations are more strongly related to moral behavior in contexts where such behavior is explicitly expected. Specifically, we compared OUS-DE scores with self-reported agreement to (moral judgment) and compliance with (moral behavior) quarantine rules that required individuals to forgo certain personal needs for the benefit of society.

We expected that individuals scoring high on the OUS-DE would report greater agreement with, and higher compliance to, government quarantine regulations, both in general and during the Christmas and New Year period. We also hypothesized that these regulations could appeal to two distinct groups: those who prioritize adherence to moral norms (e.g., high deontological, low utilitarian agreement) and those who aim to maximize the greater good (high utilitarian agreement), due to their rule-based structure and utilitarian content, respectively [78,83]. Accordingly, we tested both linear and quadratic (U-shaped) associations between OUS-DE scores, self-reported moral judgement, and moral behavior regarding adherence to quarantine rules.

Our second aim was to extend the findings of Study 2 by examining changes in utilitarian inclinations in women and men over the course of the Covid-19 pandemic. For this purpose, we performed repeated measurement of OUS-DE at two time points: during the first year of the pandemic (October-December 2020) and towards its end (March 2023). As in Study 2, we expected higher agreement with Utilitarianism, especially with the IH dimension.

### Materials and methods

#### Sample description.

Fifty-eight participants took part in a separate laboratory study on moral decision-making and the influence of sex hormones between October – December 2020. Exclusion criteria were neurological or mental disorders, severe hypertension, diabetes, thyroid diseases, congestive heart failure, vasectomy, use of hormonal contraceptives, hormonal treatment, premenstrual dysphoric disorder, current pregnancy and birth or breastfeeding within the last year. By the end of December 2020, participants of this study were invited to complete a supplemental battery of questions regarding Covid-19 regulations. Of all 58 participants invited, 41 participants completed the online survey during the period between 2020-12-15 and 2021-01-10. Two participants were excluded as they did not fulfil the criterion of spending Christmas or New Year’s Eve in Germany. The final sample comprised 39 participants, of which 19 were women (*M*_age_ = 22.89, *SD* = 3.05) and 20 were men (*M*_age_ = 24.15, *SD* = 3.07), more details can be found in Table 11.

**Table 11:** Sociodemographic Characteristics of Participants after Exclusions in Study 2.

Baseline characteristic	Sample 3 (N = 39)	
	n	%
*Sex/gender*		
Women	19	48,7%
Men	20	51,3%
*Age*		
18-20	5	12,8%
21-25	25	64,1%
26-30	8	20,5%
31-35	1	2,6%
*Highest education level*		
Secondary	4	10,3%
General (high school)	27	69,2%
At least one higher education degree	8	20,5%
*Religion*		
Christianity	30	76,9%
Atheism	7	17,9%
Other	2	5,2%
*Political ideology*		
Marxist/socialist	2	5,1%
Liberal	12	30,8%
Ecologist	12	30,8%
Social democratic	9	23,1%
Conservative	2	5,1%
Other	2	5,1%

For repeated OUS-DE measurement we invited all 39 participants from the final sample in early 2023 when the last Covid-19 measures (compulsory face mask wearing in public transportation and hospitals) were being phased out from daily life. Between 2023-03-06 and 2023-03-21 30 participants (N_women _= 14) completed repeated measurements of the online OUS-DE and questions regarding their and their immediate family’s history of Covid-19 infection. Twenty-five of these responders were infected with Covid-19, one suffered from “Long Covid Syndrome”, three had a close family member hospitalized because of Covid-19 infection, four had a close family member suffering from “Long Covid Syndrome”, and none had lost a close family member to Covid-19 infection.

#### Materials.

The same questionnaires were used in Study 1 and 2 (for descriptions see Materials sections in Study 1 and Study 2) with one alteration concerning the Temperament and Character Inventory (TCI). In Study 2, the whole TCI (240 items) was applied. TCI consists of two dimensions – Temperament and Character [93]. Temperament dimension involves 4 scales: Novelty Seeking (NS), Harm Avoidance (HA), Reward Dependence (RD), and Persistence (P); while Character dimension includes 3 scales: Self-directedness (S), Cooperativeness (C), and Self-transcendence (ST). All scales except for the Persistence scale are further separated into several related subscales. Higher scores on the whole scale indicate higher novelty seeking, harm avoidance, reward dependence, persistence, self-directedness, cooperativeness, and self-transcendence.

### Moral judgment and moral behavior during Covid-19 pandemic

To better understand the attitudes and motivation to comply with Covid-19 regulations in December 2020, we asked participants to provide information on whether they had tested positive for Covid-19 before, which federal German state they currently reside in, whether they live alone or with their partner/parents/in a shared flat (differentiating between < 5 and > 5 residents) and in which federal state they plan to spend/had spent Christmas (December 24^th^-26^th^) and New Year’s Eve (December 31^st^-January 1^st^), as government regulations differed slightly between states.

Participants’ attitudes – i.e., their moral judgment – towards the mandatory wearing of face masks and the enforced curfew were then assessed (all questions on Visual Analogue Scales from 0 – *not strict enough* or *not at all*, depending on the question to 100 – *too strict* or *totally*). Participants were also instructed to indicate how satisfied they are with the government’s current regulations and to what extent they are planning to/have complied with these regulations, to assess their moral behavior. Questions were based on the official regulations of Baden-Württemberg state in Germany [171]. Summary of regulations in English can be found in S2 Appendix.

For analysis purposes, responses to questions assessing personal attitudes to face mask wearing, curfew, and meeting restrictions, as well as satisfaction with general and celebration-specific regulations to prevent spread of Covid-19 (a total of 8 questions), were combined into a mean score measuring Moral Judgment (MJ). Responses to questions to what extent the participants comply with the regulations regarding face mask wearing, curfew, and celebration-specific meeting restrictions (in total 5 questions) were combined into a mean score measuring general Moral Behavior (MB) during the pandemic.

Further, it was assessed how reasonable participants consider the government’s regulations for social gatherings on Christmas and New Year’s Eve (see S1 Appendix) and to what extent they comply/complied with the regulations, to assess their moral judgment and moral behavior during Christmas and New Year.

Subsequently, participants stated the amount of people they plan to spend/have spent Christmas and New Year’s Eve with, how many of those were particularly vulnerable to infections, how many households this corresponded with, and if they plan or have taken any precautionary measures to protect the others around them, to assess their moral behavior during Christmas and New Year. Finally, participants stated whether and when they plan to get vaccinated against Covid-19.

Questions regarding precautionary measures the person was planning or had taken during the festivities were combined into moral behavior scores separately for Christmas and New Year celebrations (MB during Christmas – 21 items, or MB during New Year – 12 items). For each response indicating that the participant was planning to/ had complied with regulations or took a precautionary measure, 1 point was added. For each incompliance, 1 point was subtracted from the final score. In the end, the sum scores for MB during Christmas ranged between −13–15 points. The scores for MB during New Year’s ranged between −7–9 points.

#### Repeated measurement.

OUS-DE was assessed again in March 2023. Additionally, at this time point we asked participants to self-report whether they believe that their moral inclinations and moral behavior have changed during the pandemic (1 question each) and provide other Covid-19 related demographic information, such as whether the participant was ever infected with Covid-19, hospitalized because of Covid-19, suffered or still suffers from “Long Covid Syndrome” and whether the participant had a close family member who hospitalized because of Covid-19, suffered or still suffers from “Long Covid Syndrome”, or died because of Covid-19.

#### Procedure.

The Ethics commission of the Medical Faculty at the University of Tübingen approved the study (“Modulatory effects of sex hormones on moral decision-making”, project number 707/2019BO2). By the end of December 2020, all participants that had taken part in the separate laboratory study on moral decision-making until this point were contacted directly via E-Mail whether they would like to participate in a subsequent survey on moral decision-making in times of Covid-19.

To assess participants’ attitude towards strict Covid-19 regulations that were in place in Germany between in December 2020 and January 2021, questions described in Materials section of this study were administered online via SoSciSurvey [89]. Participants were instructed to answer all questions truthfully, even if their true answer should not adhere to the government’s regulations. To further lower concerns, they were reminded that all collected data was pseudonymized and could not be traced back. Participants were compensated for taking part in a separate laboratory study on moral decision-making.

All participants, which had answered the subsequent questions on their attitude and compliance with Covid-19 restrictions in December 2020 – January 2021 and gave consent to be contacted again for further research purposes, were again invited by E-Mail in 2023.03.07–2023.03.20 to fill out the repeated measurement of OUS-DE via SoSciSurvey, the time period when the last German pandemic measures were being phased out. Written informed consent was obtained from the participants. Participants were compensated 5 Euros for participation in this survey. The data was stored in a pseudonymized form. This study was not preregistered.

### Data analysis

#### Predictive associations.

To examine predictive relationships, linear regression models were constructed with moral judgment (MJ), moral behavior (MB), MB during Christmas, and MB during New Year as dependent variables and individual OUS-DE, IB-DE, and IH-DE scores as predictors. To test for potential nonlinear relationships, nonlinear regression models were additionally applied.

#### Linear associations.

Linear associations between MJ, MB, MB during Christmas, and MB during New Year were investigated by using bivariate Pearson’s two-tailed correlations. Correlation strength was interpreted as in Study 2 (please refer to Data analysis section for more information).

#### Repeated measures.

Two-way repeated measures ANOVA was applied to investigate variance of OUS-DE and its subscales over time (2020, 2023) as a within-subject factor and sex/gender as a between-subjects factor as sex/gender was found to have an independent effect on the changes in agreement with IB-DE in Study 2. This model allowed us to examine both main effects (time, sex/gender) and their interaction effects on individual OUS-DE scores and subscale scores.

### Results: Study 3

#### OUS-DE measurement during the Covid pandemic.

In this sample (N = 39), mean agreement with the whole OUS-DE measurement was highly comparable to the mean responses in both samples from Study 1 (see Table 12). Correlation analysis indicated that the OUS-DE score strongly correlated with the scores in the two subscales – IB-DE (r = 0.788, p < .001) and IH-DE (r = 0.534, p < .001), similarly to the results in Study 1.

**Table 12:** Mean responses to the OUS-DE, its subscales, quarantine moral judgement, quarantine moral behavior, and moral behavior during Christmas and New Year in Sample 3.

	Sample 3 Mean score (range)
**OUS-DE**	3.91 (2.22–5.44)
**IB-DE**	4.37 (1.8–6.2)
**IH-DE**	3.22 (1.0–5.75)
**Moral judgment (MJ)**	67.57 (21.75–95.13)
**Moral behavior (MB)**	89.28 (63.4–101.0)
**MB during Christmas**	4.1 (−30–60)
**MB during New Year**	10.51 (−30–60)

#### Relationship between OUS-DE and moral judgment and behavior during Covid-19 pandemic.

**Fig 3:**
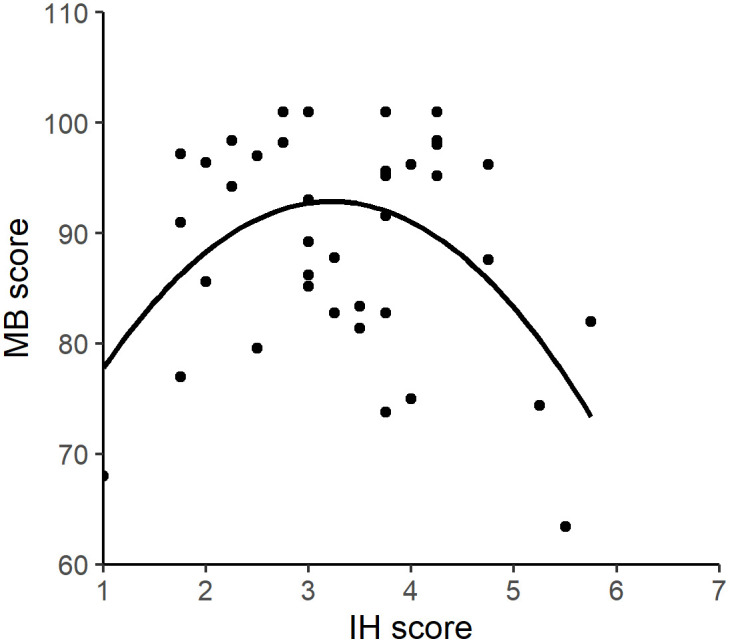
Quadratic inverse U-shape relationship between Moral Behavior during the pandemic and Instrumental Harm subscale.

We found no predictive linear relationship between OUS-DE and its subscales and MJ or MB (all p’s > .09). Nonlinear regression analysis indicated that there was an inverse U-shape quadratic relationship between general MB and IH-DE subscale (R^2^ = 0.23; β = −3.05, p = .003; F(2, 430.65) = 5.377, p = .009, as also depicted in Fig 3). No linear or nonlinear relationships with OUS-DE or its subscales were found for MJ, MB during Christmas, and MB during New Year (all p’s > .11).

Further, MB and MJ scores correlated positively and moderately strongly with each other (r = 0.55, p < .001). MB during Christmas positively and moderately correlated with MB during New Year (r = 0.5, p = .001). MJ and MB did not correlate with MB during either Christmas or New Year. For these correlations, please see Table 13.

**Table 13:** Correlations between the OUS-DE, its subscales, quarantine moral judgement, quarantine moral behavior, and moral behavior during Christmas and New Year.

	OUS-DE	IB-DE	IH-DE	MJ	MB	MB-Christmas
OUS-DE	–					
IB-DE	.79**	–				
IH-DE	.53**	−0.1	–			
MJ	−0.18	−0.02	−0.27	–		
MB	−0.1	−0.02	−0.13	.55**	–	
MB-Christmas	−0.12	−0.17	0.04	0.25	0.15	–
MB-New Year	0.05	−0.13	0.26	0.01	0.06	.5**

** Correlation is significant at the 0.01 level (2-tailed).

We have additionally investigated sex/gender differences for MJ and MB measurements in this study. No sex/gender differences were observed for any of the scores (all p’s > .39, see S1 Table).

### Peri- and post-pandemic OUS-DE responses.

A two-way repeated measures ANOVA was used to assess whether responses to OUS-DE and its subscales changed with time (within-subjects factor) in women and men (between-subjects factor). We observed no significant time effect on OUS-DE (F(1, 28) = 0.212, p = .65), IB-DE subscale (F(1, 28) = 0.414, p = .525), and IH-DE subscale (F(1, 28) = 0.032, p = .86). Time and sex/gender interaction was, too, not significant in the OUS-DE and its two subscales when analyzed separately (all p’s > .073). Assessment of between subject effects did not find sex/gender differences for OUS-DE (F(1, 28) = 3.23, p = .083) or IH-DE (F(1, 28) = 0.031, p = .86) ratings. Sex/gender effect in ratings of IB-DE reached a trend level towards significance (F(1, 28) = 4.16, p = .051).

Additionally, we asked study participants during repeated measurement of OUS-DE to self-report whether their moral views or moral behavior have changed after the pandemic. 6.7% indicated that their moral views have not changed at all, 53.3% indicated that they have changed a little, 26.7% indicated that they have changed a lot, and 13.3% indicated that they have changed significantly. In a separate question, 23.3% reported that their moral behavior had not changed at all, 30% reported that it had changed a little, 40% reported that it had changed a lot, and 6.7% reported that it had changed significantly.

## Discussion: Study 3

### Relationship between utilitarian inclinations and pandemic-related moral judgment and behavior.

The primary goal of Study 3 was to investigate whether utilitarian inclinations were associated with self-reported moral judgment of pandemic safety measures and moral behavior as expressed by adherence to these measures during the first winter of the Covid-19 pandemic. Contrary to our expectations, we found an inverted U-shape relationship between agreement with the IH-DE subscale and conformity to the quarantine rules (MB score), indicating that those scoring the lowest and the highest were least conforming to the quarantine rules, such as mask wearing, distance keeping, and limitation of social contacts. We also found that agreement with quarantine rules and conformity to those rules positively correlated to each other but not to conformity to quarantine rules during Christmas and New Years celebrations. Finally, we observed that behavior scores during the festivities positively correlated. Overall, our results indicate that participants in this study based their judgments and behavior towards conformity to the quarantine rules on circumstantial principles (i.e., important traditions) or their specific agreement with the quarantine rules but not their general utilitarian inclinations.

Our results in this study are in line with some recent findings from the general population as well as healthcare staff, which show that not moral inclinations but rather sociodemographic and circumstantial variables are more closely related to moral behavior during the pandemic. Lachowicz-Tabaczek and Kozłowska [172] assessed social distancing behavior in 505 Polish participants from the general public and found that it was predicted by concern for collective health and personal values of social responsibility, although they did not assess utilitarian inclinations in this sample. In a sample of 49 Iranian women medical professionals, age, number of encountered deaths from Covid-19, perceived self-risk of infection, and stress level but not utilitarian inclinations as measured by sacrificial moral dilemmas positively correlated with social distancing behavior [27]. In an online survey with 197 participants from the general public, Díaz [86] showed that actual past and future compliance to Covid-19 regulations was better predicted by moral emotions that arise in response to moral statements involving harm than factual moral beliefs’ statements involving harm. Similarly, in a Dutch general public sample (N = 1396), moral values as measured by the MFQ were weak predictors of conformity to Covid-19 regulations, while other sociodemographic variables, such as gender and age had better predictive power [84].

There is also some evidence that utilitarian inclinations might have been associated with decisions in experimental pandemic-related triage dilemmas. Kneer and Hannikainen [123] relied on an US sample of around 1100 participants from the general public and reported that the IB but not the IH subscale scores predicted answers to realistic pandemic-related triage dilemmas. In a large sample of over 15000 individuals from countries in Latin America (mostly Argentina) and a separate sample from the US (N = 1300), Navajas [25] observed that commitment to Utilitarianism as measured by sacrificial harm dilemmas and both OUS subscales in an online survey modulated moral decisions regarding the Covid-19 pandemic in triage dilemmas. In this study, prioritized treatment for younger over older patients was positively associated with IH subscale scores, while priority to equitable public health was positively associated with IB subscale scores, and both scales were positively associated with preference to public health over data-privacy, economic activity, and friendship.

Taken together, results from our study as well as previous research indicate that when assessing actual pandemic-related behavior, other personal and situational variables, such as social orientation, emotional response to harm, age, stress, or number of encountered deaths from Covid-19 are better predictors of the behavior than general utilitarian inclinations. Associations between the two dimensions of Utilitarianism could only be found in experimental studies assessing behavior in triage situations but not in conformity to the quarantine rules directed at the general public. This might suggest that either a methodological effect exists in assessing experimental scenarios even if they are realistic, or that utilitarian inclinations play a role in triage situations only, as both assessments deal with extreme sacrifice.

## General discussion

The main aim of the paper was to validate the German translation of the Oxford Utilitarianism Scale (OUS-DE). The studies in this paper show psychometric robustness and construct validity of OUS-DE and provide evidence of reliability of the translated version for future research. In Study 1, we demonstrated moderate to strong linear associations between utilitarian inclinations as measured by OUS-DE subscales and choice preferences in thematically corresponding moral dilemmas. In Study 2, we confirmed conceptual associations between Impartial Beneficence (IB) and empathy as well as concern for Harm/Care foundation. Study 3, maybe surprisingly, revealed that association of utilitarian inclinations regarding instrumental harm or behavioral beneficence are not linearly related to actual moral behavior during the Covid-19 pandemic. Nevertheless, our analyses of OUS-DE responses before, during, and after the Covid-19 pandemic in Studies 2 and 3 indicate that the questionnaire captures stable utilitarian inclinations in a young German sample. In two studies we also found a consistent sex/gender difference regarding responses to IB-DE, with women accepting IB to a higher degree, independently from the measurement time point.

Across the studies, we have consistently observed a context-dependent role of Utilitarianism. As measured by the OUS-DE, similarly to measurements by high-stakes sacrificial scenarios, utilitarian beliefs remained stable. However, when the public discourse and policy repeatedly invoked requirements for utilitarian solutions, endorsement of IB shifted. Finally, the weak translation of utilitarian inclinations into everyday compliance behaviors further strengthened the assumption of the context dependence. These findings highlight the importance of separating moral belief endorsement from moral behavior in future research, the so-called “moral judgment-behavior gap” [173]. Increasing evidence is provided by studies testing the existence of this gap in other contexts rather than Covid-19 pandemic, for example, impartial resource allocation [173,174] or political voting behavior [127].

Our findings also suggest a need for longitudinal designs to track the changes in individual moral inclinations. Current turbulent geopolitical and societal events might be uniquely reshaping moral beliefs in the general population as well as specific professional groups such as healthcare workers, public servants, or military personnel. Future investigations could see how changes or stability in personal moral beliefs relate to the changing values around national sovereignty, identity, and community [175].

On a more methodological note, our studies showed that there might be potential for conceptual overlap between items in the OUS-DE. While no other validation studies have encountered this issue, culturally sensitive adaptations of OUS could be considered. Furthermore, our studies also show that the conceptual associations between the two dimensions of Utilitarianism should be further clarified as they might operate through different cognitive-affective pathways yet culminate into one coherent moral outlook. Exploration of a “dual-high” utilitarian profile – high on IB and high on IH – and its cognitive-affective correlates would aid in this pursuit. Finally, future studies should further investigate in which contexts the OUS – and self-reported moral inclinations in general – has a high predictive validity and where the moral judgment-behavior gap persists.

Taken together, OUS-DE is a valid and reliable tool to assess utilitarian moral inclinations in German-speaking populations. The degree to which an individual’s moral judgements cohere with Utilitarianism is of interest to moral psychology and beyond. The recent Covid-19 pandemic has brought to the forefront the inevitability of triage and other cost-benefit analyses in healthcare. Some reported experiencing a ‘moral injury’ during the pandemic, having to behave in ways that conflict with their moral beliefs, such as when denying help to incoming patients in overcrowded hospitals during the most intense Covid-19 periods [176–178]. Knowing the folk attitude to Utilitarianism can help in understanding individual responses to such dilemmas, in gauging the expected agreement to utilitarian policies, in informing public decision-making, and in designing interventions for moral resilience in healthcare and policy.

## Limitations

Some limitations of our validation study should be noted. First, our samples mainly consist of young adults, with the majority studying at university. The surveys were advertised in the University town of Tübingen, a city with one of the youngest average populations in Germany [179]. Therefore, our results might not generalize to the general German population and future studies should assess OUS-DE in older populations, as age effects had been shown in moral inclinations [71]. Our validation study was also performed online, which might hinder reliability of responses to the survey. We have implemented a minimum time threshold for completion of the survey and rigorous univariate and multivariate outlier analyses to identify any sporadic responses to the survey; however, we are not able to check the sincerity of the survey answers, a problem that any self-report measure would face. Finally, in Study 1 we did not perform a repeated measures survey pre- and post-pandemically, as it was impossible due to the anonymous nature of our survey. Notwithstanding these limitations, our validation and cross-validation studies demonstrate robust validity and reliability properties of the OUS-DE, comparable to the original English version.

In Study 2, we have compared measurements of OUS-DE between two different samples when investigating the effects of Covid-19 pandemic. While this was necessary due to the anonymous nature of our online surveys, the observed sample effect in the difference of agreement to IB-DE subscale might be due to other reasons rather than the pandemic, for example, different but unaccounted sociodemographic constitution of the samples or a societal shift in moral attitudes due to other historical events. As we are aware of this limitation, we have added an alternative explanation for sample effects in the discussion of Study 2.

The sample size in Study 3 might be considered relatively small and not generalizable to the general population, as all participants in the study were young adults. As this study was part of a larger in-person experiment, due to the Covid-19 pandemic, collection of data was limited and resulted in a small sample size.

Notwithstanding these limitations, sample sizes and their sociodemographic constitution is comparable to many studies in the field of psychology, while our validation study demonstrates robust validity and reliability properties of the OUS-DE, comparable to the original English version.

## Conclusion

The present research provides a comprehensive validation of the German translation of the Oxford Utilitarianism Scale (OUS-DE). Across three studies, we have demonstrated this translation’s psychometric robustness, construct validity, and reliability and supported its conceptual consistency with the original version. This establishes OUS-DE as a sound tool for assessing utilitarian moral inclinations in German-speaking populations.

Our research also highlighted important new findings such as a consistent sex/gender difference in endorsement of impartial beneficence and possible influence of historical events on agreement with individual items in the subscales. Moreover, our results support the findings of other studies underscoring that self-reported moral inclinations do not directly translate into moral behavior. This empathizes with the need to further investigate a well-documented moral judgment-behavior gap in the context of utilitarian morality. This has significant implications for applied fields such as healthcare, policy-making, and moral education, particularly in contexts where individuals are required to make high-stakes moral decisions, as seen during the Covid-19 pandemic.

Future studies might utilize the OUS-DE in assessing general utilitarian beliefs in the general population and in specialized populations. OUS-DE could be also used as an experimental or as a diagnostic tool to identify vulnerable populations when the circumstances require strict behavior in accordance with a particular moral theory. Finally, it could also be assessed in sociological investigations and opinion polls related to agreement with various policies or political support. Sex/gender differences in impartial beneficence should be considered when the measurement is applied.

In sum, the OUS-DE is a reliable and valid instrument that not only advances research in moral psychology but also provides a valuable foundation for applied studies aiming to understand and support moral reasoning in times of societal, political, or global crises.

## Supporting information

S1 File OUS-DE confirmatory factor analysis (CFA) in women and men (Sample 1) separately. Data analysis description, factor loadings for OUS-DE in women and in men separately, CFA results in women and in men separately.
(DOCX)

S1 Appendix Pen and paper style German Oxford Utilitarianism Scale. (PDF)

S2 Appendix English summary of Covid-19 regulations in Baden-Württemberg state, Germany, where the survey was conducted. Link to the original regulations in German and a summary translation to English.
(DOCX)

S1 Table Sex differences in quarantine related moral judgment (MJ), moral behavior (MB), and moral behavior (MB) during Christmas and New Year in Sample 3. Results of t-test analyses in a table.
(DOCX)
