# Supplementary material for: The German translation of the Oxford utilitarianism scale: Validation and the impact of the Covid-19 pandemic on the observations
Source: PLoS One. 2025 Oct 27;20(10):e0335215. doi: 10.1371/journal.pone.0335215 (PMC12558481; doi:10.1371/journal.pone.0335215)
Supplement: S1 File — Data analysis description, factor loadings for OUS-DE in women and in men separately, CFA results in women and in men separately. (DOCX) [file pone.0335215.s001.docx]

**S1 File. OUS-DE confirmatory factor analysis in women and men (Sample 1) separately**

**Supplementary Note A.** Confirmatory factor analysis by sex groups *p. 2*

**Supplementary Table A.** Standardized factor loadings for OUS-DE in women (N = 243). p. 3

**Supplementary Table B.** Standardized factor loadings for OUS-DE in men (N = 135). p. 4

**Supplementary Table C.** CFA results in women groups of Sample 1. p. 5

**Supplementary Table D.** CFA results in men groups of Sample 1 . p. 6

**Supplementary Note A.** Confirmatory factor analysis by sex groups

Confirmatory factor analyses were performed on Sample 1 separately for women (N = 243) and men (N = 135), no data was missing. Two fixed factors were assessed based on the original questionnaire subscales, and covariance was drawn between the two factors. CFAs were based on Maximum Likelihood Estimation (7 iterations in male group of Sample 1). Results of the analysis are shown in Supplementary Table 1 and Supplementary Table 2.

The default two-factor model fell just short of adequate in both samples, and modification indices suggested that the model could be improved if covariances were modelled between questionnaire items IB-1 and IB-5 in males when the whole scale is considered, between items IB-1 and IB-5 in males when only IB-DE subscale is considered.

Modification women: between items IB-1 and IB-5 as well as IB-2 and IB-3 in women of Sample 1 when the whole scale is considered; between items IB-1 and IB-5 as well as IB-2 and IB-3 in women of Sample 1 when only IB-DE subscale is considered.

**Supplementary Table A.** Standardized factor loadings for OUS-DE in **women** (N = 243).

|  | **Sample 1** | | | |
| --- | --- | --- | --- | --- |
| Item | Default model | | Modified model | |
| IB-1 | .554 |  | .550 |  |
| IB-2 | .603 |  | .456 |  |
| IB-3 | .664 |  | .538 |  |
| IB-4 | .284 |  | .328 |  |
| IB-5 | .568 |  | .618 |  |
| IH-1 |  | .729 |  | .728 |
| IH-2 |  | .404 |  | .404 |
| IH-3 |  | .750 |  | .751 |
| IH-4 |  | .842 |  | .842 |

**Supplementary Table B.** Standardized factor loadings for OUS-DE in **men** (N = 135).

|  | **Sample 1** | | | |
| --- | --- | --- | --- | --- |
| Item | Default model | | Modified model | |
| IB-1 | .613 |  | .537 |  |
| IB-2 | .689 |  | .732 |  |
| IB-3 | .731 |  | .757 |  |
| IB-4 | .523 |  | .514 |  |
| IB-5 | .615 |  | .538 |  |
| IH-1 |  | .719 |  | .721 |
| IH-2 |  | .423 |  | .423 |
| IH-3 |  | .818 |  | .819 |
| IH-4 |  | .853 |  | .850 |

**Supplementary Table C.** CFA results in **women**

|  | | **χ2** | **TLI** | **CFI** | **SRMR** | **RMSEA** | **AIC** | **BIC** |
| --- | --- | --- | --- | --- | --- | --- | --- | --- |
| **Women in**  **Sample 1** | Two-factor default model | 61.928, df = 26, p< 0.001 | 0.896 | 0.925 | 0.0613 | 0.076 | 99.928 | 166.296 |
|  | Two-factor modified model | 35.829, df= 25, p< 0.03 | 0.963 | 0.975 | 0.0531 | 0.045 | 77.829 | 151.183 |
|  | IB subscale defaut model | 30.290, df= 5, p< 0.001 | 0.719 | 0.859 | 0.0621 | 0.145 | 50.290 | 85.221 |
|  | IB subscale modified model | 3.407, df= 3,  p = 0.333 | 0.992 | 0.992 | 0.0211 | 0.024 | 27.407 | 69.323 |
|  | IH subscale default model | 4.017, df= 2, p= .134 | 0.979 | 0.993 | 0.0224 | 0.065 | 20.017 | 47.962 |

**Supplementary Table D.** CFA results in **men**.

|  | | **χ2** | **TLI** | **CFI** | **SRMR** | **RMSEA** | **AIC** | **BIC** |
| --- | --- | --- | --- | --- | --- | --- | --- | --- |
| **Men in**  **Sample 1** | Two-factor default model | 48.665, df = 26, p = 0.005 | 0.911 | 0.936 | 0.0824 | 0.081 | 86.665 | 89.730 |
|  | Two-factor modified model | 39.911, df= 25, p = 0.03 | 0.939 | 0.958 | 0.0788 | 0.067 | 79.911 | 138.016 |
|  | IB subscale defaut model | 11.263, df= 5, p = 0.046 | 0.919 | 0.959 | 0.0444 | 0.097 | 31.263 | 60.316 |
|  | IB subscale modified model | 3.256, df= 4,  p = 0.516 | 1.012 | 1.000 | 0.0268 | 0.000 | 25.256 | 57.214 |
|  | IH subscale default model | 1.635, df= 2,  p = 0.441 | 1.006 | 1.000 | 0.0170 | 0.000 | 17.635 | 40.878 |
