# Supplementary material for: The German translation of the Oxford utilitarianism scale: Validation and the impact of the Covid-19 pandemic on the observations
Source: PLoS One. 2025 Oct 27;20(10):e0335215. doi: 10.1371/journal.pone.0335215 (PMC12558481; doi:10.1371/journal.pone.0335215)
Supplement: S1 Appendix — (PDF) [file pone.0335215.s002.pdf]

Datum ___ / ___ / ___ (Tag / Monat / Jahr)

VP-Code: ________________________

OUS

In diesem Fragebogen werden wir Sie nach Ihrer Meinung zu verschiedenen Behauptungen über Moral fragen. Einige von Ihnen werfen schwierige Fragen auf, über die Menschen unterschiedlicher Meinung sein können. Wir suchen nach keinen bestimmten Antworten oder versuchen zu sehen, wie moralisch Menschen sind. Wir sind nur an Ihrer ehrlichen Meinung zu diesen Themen interessiert.

Bitte lesen Sie jede Frage aufmerksam durch und beantworten Sie sie. Bitte versuchen Sie jegliche Ablenkung abzuschalten und sich auf die Fragen zu konzentrieren.

Wie sehr stimmen Sie den folgenden Aussagen zu?

		Stimme gar nicht zu	Stimme nicht zu	Stimme eher nicht zu	Teils-teils	Stimme eher zu	Stimme zu	Stimme voll und ganz zu
1	Unter moralischen Gesichtspunkten sollten sich Menschen gleichermaßen um das Wohlergehen aller Menschen auf dem Planeten sorgen. Sie sollten das Wohlergehen der Personen, die ihnen physisch oder emotional nahe stehen, nicht bevorzugen.	☐	☐	☐	☐	☐	☐	☐
2	Von einem moralischen Standpunkt aus gesehen sollten wir uns verpflichtet fühlen, eine unserer Nieren an eine Person mit Nierenversagen zu geben. Denn wir brauchen nicht zwei Nieren um zu überleben, sondern nur eine, um gesund zu sein.	☐	☐	☐	☐	☐	☐	☐
3	Wenn in einem Notfall die einzige Möglichkeit, das Leben einer anderen Person zu retten, darin besteht, sein eigenes Bein zu opfern, dann ist dieses Opfer moralisch geboten.	☐	☐	☐	☐	☐	☐	☐
4	Es ist genauso falsch, jemandem Hilfe zu versagen, wie jemandem selbst aktiv zu schaden.	☐	☐	☐	☐	☐	☐	☐
5	Es ist moralisch falsch, Geld zu behalten, welches man nicht wirklich braucht, statt es Zwecken zu spenden die effektive Hilfe für jene bieten, die daraus großen Nutzen ziehen.	☐	☐	☐	☐	☐	☐	☐
6	Es ist moralisch richtig, einer unschuldigen Person zu schaden, wenn dieser Schaden ein notwendiges Mittel ist, mehreren anderen unschuldigen Personen zu helfen.	☐	☐	☐	☐	☐	☐	☐

7	Wenn die einzige Möglichkeit den Menschen allgemeines Wohlergehen und Glück zu ermöglichen die Nutzung politischer Unterdrückung für eine kurze, begrenzte Zeit ist, dann sollte politische Unterdrückung genutzt werden.	☐	☐	☐	☐	☐	☐	☐
8	Wenn es für die Entschärfung einer Bombe, die hunderte Menschen töten würde, notwendig ist, eine unschuldige Person zu foltern, ist dies moralisch zulässig.	☐	☐	☐	☐	☐	☐	☐
9	Manchmal ist es moralisch notwendig, dass unschuldige Menschen als Kollateralschaden sterben – wenn dadurch insgesamt mehr Menschen gerettet werden.	☐	☐	☐	☐	☐	☐	☐
