# Supplementary material for: The German translation of the Oxford utilitarianism scale: Validation and the impact of the Covid-19 pandemic on the observations
Source: PLoS One. 2025 Oct 27;20(10):e0335215. doi: 10.1371/journal.pone.0335215 (PMC12558481; doi:10.1371/journal.pone.0335215)
Supplement: S2 Appendix — Link to the original regulations in German and a summary translation to English. (DOCX) [file pone.0335215.s003.docx]

**S1 Appendix.** English summary of Covid-19 regulations in Baden-Württemberg state, Germany, where the survey was conducted.

Original document: https://www.baden-wuerttemberg.de/fileadmin/redaktion/dateien/PDF/Coronainfos/201215_CoronaVO_konsolidierte_Fassung_ab_201216.pdf

English summary: Citizens and visitors in Baden-Württemberg were generally required to wear protective face masks in public buildings/the supermarket and in public spaces outside/the city centre when distance of 1.5 meters could not be sustained; meetings were allowed for maximum 5 individuals (except children) from 2 households; curfew from 20:00 to 05:00. For period of 2020-12-24 – 26, meeting with 4 more immediate family members from multiple households was allowed, for non-related individuals 5 people from 2 households rule applied; curfew relaxed; travel to celebration location allowed also by train. For New Years Eve no changes in general distancing or meeting rules were applied.
