# Supplementary material for: The German translation of the Oxford utilitarianism scale: Validation and the impact of the Covid-19 pandemic on the observations
Source: PLoS One. 2025 Oct 27;20(10):e0335215. doi: 10.1371/journal.pone.0335215 (PMC12558481; doi:10.1371/journal.pone.0335215)
Supplement: S1 Table — Results of t-test analyses in a table. (DOCX) [file pone.0335215.s004.docx]

**S1 Table.** Results of t-test comparing MJ, MB, MB during Christmas and MB during New Year in women and men in Sample 3.

**Supplementary Table 5.** Sex differences in quarantine related moral judgment (MJ), moral behavior (MB), and moral behavior (MB) during Christmas and New Year in Sample 3.

|  | **Sex/gender** | | | |  | | |
| --- | --- | --- | --- | --- | --- | --- | --- |
|  | Women (N = 19) | | Men (N = 20) | | Mean difference | | |
|  | *M* | *SD* | *M* | *SD* | t(37) | *p* | Cohen’s d |
| MJ | 68.67 | 12.63 | 66.53 | 18.19 | 0.425 | .67 | 0.136 |
| MB | 89.35 | 9.53 | 89.22 | 10.54 | 0.04 | .97 | 0.013 |
| MB during Christmas | 7.89 | 22.26 | 13.0 | 18.09 | -0.788 | .44 | 0.252 |
| MB during New Year | 6.32 | 17.07 | 2.0 | 13.99 | 0.866 | .39 | 0.277 |
